# Exosomal miR-218-5p/miR-363-3p from Endothelial Progenitor Cells Ameliorate Myocardial Infarction by Targeting the p53/JMY Signaling Pathway

**DOI:** 10.1155/2021/5529430

**Published:** 2021-07-16

**Authors:** Xiao Ke, Rongfeng Yang, Fang Wu, Xing Wang, Jiawen Liang, Xun Hu, Chengheng Hu

**Affiliations:** ^1^Department of Cardiology, Fuwai Hospital, Chinese Academy of Medical Sciences, Shenzhen (Shenzhen Sun Yat-sen Cardiovascular Hospital), Shenzhen, Guangdong 518057, China; ^2^Shenzhen University School of Medicine & Shenzhen University Health Science Center, Shenzhen, Guangdong 518060, China; ^3^Department of Cardiology, The Eighth Affiliated Hospital, Sun Yat-sen University, Shenzhen, Guangdong 518033, China; ^4^Key Laboratory on Assisted Circulation, Ministry of Health, The First Affiliated Hospital of Sun Yat-sen University, Guangzhou, Guangdong 510080, China; ^5^Department of Geriatric, The First Affiliated Hospital of Sun Yat-sen University, Guangzhou, Guangdong 510080, China; ^6^Department of Cardiology, The First Affiliated Hospital of Sun Yat-sen University, Guangzhou, Guangdong 510080, China

## Abstract

Accumulating evidence has shown that endothelial progenitor cell-derived exosomes (EPC-Exos) can ameliorate myocardial fibrosis. The purpose of the present study was to investigate the effects of EPC-Exos-derived microRNAs (miRNAs) on myocardial infarction (MI). A miRNA-Seq dataset of miRNAs differentially expressed between EPCs and exosomes was collected. Quantitative real-time polymerase chain reaction (qRT-PCR) was used to validate the miRNA expression indicated by miRNA-Seq. Immunofluorescence, cell proliferation, and angiogenesis assays were employed to investigate the effects of miRNAs on cardiac fibroblasts (CFs) *in vitro*. Interactions between miRNAs and their respective targets were examined via immunoblotting, qRT-PCR, and luciferase reporter assays. An MI rat model was constructed, and various staining and immunohistochemical assays were performed to explore the mechanisms underlying the miRNA-mediated effects on MI. miR-363-3p and miR-218-5p were enriched in EPC-Exos, and miR-218-5p and miR-363-3p mimic or inhibitor enhanced or suppressed CF proliferation and angiogenesis, respectively. miR-218-5p and miR-363-3p regulated p53 and junction-mediating and regulatory protein (JMY) by binding to the promoter region of p53 and the 3′ untranslated region of JMY. Additionally, treatment of CFs with Exo-miR-218-5p or Exo-miR-363-3p upregulated p53 and downregulated JMY expression, promoted mesenchymal-endothelial transition, and inhibited myocardial fibrosis. Administration of exosomes containing miR-218-5p mimic or miR-363-3p mimic ameliorated left coronary artery ligation-induced MI and restored myocardial tissue integrity in the MI model rats. In summary, these results show that the protective ability of EPC-Exos against MI was mediated by the shuttled miR-218-5p or miR-363-3p via targeting of the p53/JMY signaling pathway.

## 1. Introduction

Cardiovascular diseases account for over 40% of deaths annually and are the leading cause of death around the world [1]. Of note, chronic heart failure (CHF) is the leading cause of cardiovascular mortality, with an increased prevalence in recent years [2]. Myocardial infarction- (MI-) induced remodeling of the left ventricle (LV) is the most common cause of CHF [3]. The clinical features of MI include LV dilatation, cardiomyocyte death, and myocardial remodeling [3, 4]. Irreversible cardiac tissue damage caused by MI leads to myocardial fibrosis (MF), ventricular remodeling, cardiac dysfunction [5], and finally heart failure [6–8]. MF is the final pathological consequence of cardiovascular disease and is characterized by abnormal thickening of heart valves or excessive deposition of extracellular matrix in the cardiac muscle [9], which results in progression to heart failure [10, 11]. Therefore, alleviation or prevention of MF is an important therapeutic strategy for the management of MI.

Endothelial progenitor cells (EPCs) originate from the bone marrow, localize within damaged tissues, and play a vital role in the regeneration of the endothelial lining of damaged blood vessels during heart attacks [12], representing a possible therapeutic treatment for MI. Previous studies have reported that a higher level of circulating EPCs predicted a better outcome for patients with cardiovascular disease [13]. Small-scale clinical trials have confirmed EPCs as a potential treatment for MF [14] and MI [15, 16]. However, to date, the functional substrates and potential molecular mechanisms associated with EPC therapy for MF or MI have not been thoroughly elucidated. Previous studies have demonstrated that CFs have the potential to transform into pluripotent stem cells, myoblasts, neurons, and endothelial cells [17]. More importantly, exosomes from EPCs have the potential to promote CFs differentiated into endothelial cells by upregulating the expression of mesenchymal-endothelial transition- (MEndT-) related genes and increasing the expression of high-mobility group box 1 protein B1 [18]. However, the regulatory mechanisms underlying the role of CFs in MI remain elusive.

Exosomes are secreted by various cell types [19, 20] and contain various paracrine factors, including proteins, lipids, and RNA. Moreover, exosomes vary in diameter from 40 to 100 nm [21] and can be used to mediate intercellular communication [22]. EPC-derived exosomes (EPC-Exos) can accelerate cutaneous wound healing [23] and ameliorate acute lung injury [24], opening a new horizon for therapeutic applications of EPC-Exos. Our previous study confirmed that EPC-Exos induce proliferation and angiogenesis of CFs and provide promising therapeutic effects for MF [18].

Emerging evidence indicates that exosomes can transport functional microRNAs (miRNAs) to induce degradation of target gene messenger RNA (mRNA) [25, 26] and play significant roles in physiology and disease [27, 28]. Circulating miRNAs can also serve as putative biomarkers of MF [29]. miRNA-enriched exosomes contribute to cardiac nuclear factor (erythroid-derived 2)-like 2 (Nrf2) dysregulation and have the potential to reduce MI-related adverse events and attenuate MI-induced myocardial injury [30]. Therefore, we hypothesized that the therapeutic effects of EPC-Exos on MF or MI may be mediated by miRNAs released from EPC-Exos.

To date, there is no reported research on the therapeutic potential of miRNAs secreted by EPC-Exos for MI. In the present study, we characterized the functional miRNAs secreted from EPC-Exos and confirmed the effects of these miRNAs on the proliferation and angiogenesis potential of CFs. In addition, the biological roles of functional miRNAs were evaluated in experimental rats with MI established via left coronary artery (LCA) ligation.

## 2. Materials and Methods

### 2.1. Sequence Analysis of EPC-Exos miRNAs

Total RNA from EPCs and EPC-Exos was isolated by means of a mirVana miRNA isolation kit (Ambion, Austin, TX, USA). One biological repeat with three mixed samples for EPCs and EPC-Exos was used for the sequence analysis. The concentration and quality of RNA samples were detected with an Agilent 2100 Bioanalyzer (Agilent Technologies, Inc., Santa Clara, CA, USA), and 50 ng RNA samples were fragmented and used for miRNA sequencing on Genome Analyzer IIx (Illumina, San Diego, CA, USA). After generating the sequences, a BLASTN search was utilized to identify conserved miRNAs, and Rfam was employed (European Molecular Biology Laboratory, Heidelberg, Germany) for the deletion of non-miRNA sequences. miRDeep2 was used to predict novel miRNAs. The length distribution of the cells and exosomal miRNAs was analyzed, and miRNAs with differential expression between cells and exosomes were detected as described previously [31]. Gene Ontology (GO) categories [32] and Kyoto Encyclopedia of Genes and Genomes (KEGG) pathway analyses [33] were utilized to investigate specific functional roles of differentially expressed miRNAs. GO enrichment analysis, including biological processes, cellular component, and molecular function, identified which GO terms were over or underrepresented within a given set of genes. The KEGG knowledge database, an integrated database resource, is generally used to identify functional and metabolic pathways. To determine whether the expression changes in miR-218-5p and miR-363-3p were influenced by the number of exosomes secreted from EPCs, GW4869 (an inhibitor of neutral sphingomyelinase 2, 10 *μ*M for 12 h, MCE China) was used to treat EPCs, and then, EPC-Exos were isolated using a Total Exosome Isolation Reagent Kit (4478359, Thermo Fisher, USA).

### 2.2. Quantitative Real-Time Reverse Transcription-Polymerase Chain Reaction (qRT-PCR)

Total RNA samples were extracted from EPCs, EPCs-Exos, and tissues around an infarct using Trizol reagent (Takara, Dalian, China). The synthesis of cDNA used for gene and miRNAs was performed using a Bestar™ qPCR RT kit (DBI; #DBI-0) and Bestar™ miRNA qPCR RT kit (DBI; #2220), respectively. The relative mRNA levels of *α*-SMA, vimentin, CD31, VEGFR-2p53, JMY, and miRNAs were determined via real-time quantitative PCR using a 20 *μ*L reaction system. PCR was performed on an ABI PRISM 7300 real-time PCR system (Applied Biosystems, Carlsbad, CA, USA) using the following conditions: 95°C for 2 min, followed by 40 cycles of 94°C for 20 sec, 58°C for 20 sec, and 72°C for 20 sec. GAPDH or U6 was used as the internal reference for genes and miRNAs, respectively. Fold changes were determined via 2^−*ΔΔ*Ct^ (where ΔCt = (Ct of miRNA of interest) − (Ct of U6), and ΔΔCt = (ΔCt of miRNA of interest) − (ΔCt of U6)) for at least three biological repeats with three technological replicates. All the primer sequences used are listed in Supplemental Table 1.

### 2.3. Luciferase Reporter Assay

Through TargetScan (http://www.targetscan.org) and the UCSC (https://genome.ucsc.edu/) website, we successfully predicted the putative targets of miR-218-5p and miR-363-3p as the p53 promoter region and the 3′UTR of junction-mediating and regulatory protein (JMY), respectively. The interaction between the p53 promoter region and miR-218-5p and the 3′UTR of JMY and miR-363-5p was evaluated using a Dual-Luciferase Reporter Assay System (Promega, Madison, USA) as instructed by the manufacturer. For the p53 promoter region, wild-type and mutant sites were constructed and subcloned into pGL3-Basic vector (Promega, E1751, Madison, USA). For the 3′UTR of JMY, wild-type and mutant sites were constructed and subcloned into psi-Check2 vector (Promega, C8021, Madison, USA). Using Lipofectamine™ 2000 transfection reagent (cat. #52887; Invitrogen, Carlsbad, USA), CFs were cotransfected with pGL3-Basic or pGL3-Basic-p53 promoter together with miR-218-5p and pRL-SV40, or CFs were cotransfected with psi-Check2 or psi-Check2-3′UTR-JMY together with miR-363-3p. *Caenorhabditis elegans* cel-miR-39 mimic served as the negative control. Forty-eight hours after transfection, the cells were washed twice with phosphate-buffered saline and lysed using passive lysis buffer. Luciferase activity was evaluated with the Dual-Luciferase Reporter Assay System (Promega). The primers used for vector construction are listed in Supplemental Table 2.

### 2.4. Isolation of EPC-Exos, Cell Culture, and Transfection

The preparation and culture of EPCs and cardiac fibroblasts were described previously [18]. Briefly, for preparation and culture of EPCs, human peripheral blood (1 : 1 with phosphate-buffered saline (PBS)) was overlaid onto a separation medium (GE Healthcare, Piscataway, NJ, USA) with endothelial growth factors and cytokines. The medium was centrifuged at 2,000 r/min for 30 min, and the mononuclear cells were collected. After washing with PBS, the mononuclear cells were placed into a 25 cm^2^ culture bottle with DMEM (Dulbecco's modified Eagle's medium, GIBCO BRL, Grand Island, NY, USA) containing 10% FBS (fetal bovine serum, GIBCO). The FBS was precentrifuged to remove any exosomes (100,000 g for 3 h). After 72 h, the nonadherent cells were removed, and the medium was refreshed every three days. The cells were incubated in a 37°C constant temperature incubator with 5% CO_2_. EPCs at passages 3-6 were utilized for the following experiments. For isolation of EPC-Exos, EPCs were harvested at 90% confluency, washed with PBS three times, and cultured in EGM-2 (endothelial cell growth medium-2, Lonza, CC3162) containing 10% exosome-depleted FBS for 24 h. The culture medium from the EPCs was collected and centrifuged at 3,000 g for 30 mins, followed by 100,000 g for 90 mins at 4°C. The supernatant was centrifuged at 100,000 g for 1 h at 4°C. After that, the supernatant was discarded and the precipitation was resuspended with 500 *μ*L ice-cold PBS. After 100,000 g for 70 mins at 4°C, the supernatant was discarded, and the precipitation was resuspended in 200 *μ*L ice-cold PBS to obtain the exosomes. The morphology of the exosomes was examined via transmission electron microscopy (Hitachi H-7650, Japan). For functional assays, 10,000 EPCs (in 100 *μ*L) per well were seeded in 6-well plates for 24 h and transfected with 100 pM miRNA mimic or inhibitor of miR-6087, miR-218-5p, or miR-363-3p (synthesized by GenePharma, Suzhou, China) using Lipofectamine 2000 (Invitrogen, CA) (Supplemental Table 3), and 24 h later, the EPC-Exos were isolated, and qRT-PCR detection was performed to assess transfection efficiency. A human cardiac fibroblast cell line was purchased from PriCells (HUM-CELL-0016, Wuhan, China) and cultured in DMEM with 10% FBS to remove weakly attached and unattached cells. The CFs (1 × 10^5^ cells per well) were seeded onto 35 mm plates, cultured for three days, and then cocultured with 5 *μ*g/mL EPC-Exos [34, 35] transfected with miRNA mimic or inhibitor for 48 h before further functional assays. For p53 suppression and JMY overexpression in CFs, 1 × 10^5^ cells per well were seeded onto 35 mm plates, cultured for three days, and then transfected with 2 *μ*g si-p53 or OE-JMY and cultured for 48 h before further functional assays. si-NC and OE-NC acted as controls. In addition, the cultured CFs were transfected with 100 pM miRNA mimic or inhibitor of miR-218-5p and miR-363-3p to directly detect the role of miR-218-5p and miR-363-3p in angiogenesis and cell proliferation.

### 2.5. Cell Proliferation Assay

A volume of 100 *μ*L containing 8,000 CFs harboring si-NC, si-p53, OE-NC, or OE-JMY plasmid was cocultured with EPC-Exos in 96-well plates for 24 h, and the cells were transfected with siRNA or plasmid for another 24 h. CCK8 (Cell Counting Kit-8) assays (Solarbio, Beijing, China) were utilized to evaluate cell viability after the addition of 5 *μ*g/mL exosomes [34, 35] for another 24, 48, or 72 h. Cell viabilities were calculated via measurement of optical density at 450 nm using a spectrophotometric plate reader (BioTek, USA). Cultured cells (2 × 10^4^/mL) in a volume of 200 *μ*L were treated with 50 *μ*M BrdU (5-bromo-2′-deoxyuridine) labelling solution (B23151, Invitrogen, CA). The cells were incubated at 37°C for 2 h, fixed with 4% formaldehyde in PBS for 15 min, and permeabilized with Triton X-100 for another 20 min. Cells were incubated in 1 M HCl for 10 min on ice and neutralized with 0.1 M phosphate/citric acid buffer (pH 7.4). The cells were blocked with 0.3% Triton X-100 and 10% normal goat serum for 1 h and incubated with a monoclonal rat anti-BrdU antibody (1 : 100, Accurate) overnight at 4°C, followed by blocking with 5% BSA. Fluorescence-labelled secondary antibodies were added (1 : 1,000, Alexa Fluor 488 goat anti-rat IgG; Molecular Probes) and incubated with the cells for 1 h at room temperature. The nuclei were stained with Hoechst 33258. Immunostaining was visualized with a fluorescence microscope (Olympus inverted microscope IX71) and quantitated using flow cytometry. Cells within the field of view under the same magnification and with the same cell seeding density before the experiment were analyzed in the study.

### 2.6. Cell Phagocytosis Assay

CFs (1 × 10^4^/mL in a volume of 100 *μ*L) cocultured with EPC-Exos were seeded in a 24-well plate coated with a gelatin-based coating solution (6950, Cell Biologics) for 10 min to achieve 90% confluence. The cells were washed with 1% bovine serum albumin (BSA) and incubated in a culture medium containing 0.05% FBS at 37°C for 2 h. After removal of the cell culture medium, the cells were incubated with 10 *μ*g/mL Dil-Ac-LDL (acetylated low-density lipoprotein, labelled with 1,1-dioctadecyl-3,3,3,3-tetramethylindocarbocyanine, L-35353, Alexa 594, Life Technologies) at 37°C for 4 h. After that, the cells were incubated with anti-Dil-Ac-LDL and anti-UAE-1 (Ulex europaeus agglutinin I), and the staining was detected as described previously [18].

### 2.7. Immunofluorescence Staining

The CFs cocultured with EPC-Exos were fixed with 4% paraformaldehyde and permeabilized with 0.1% Triton X-100. After blocking with 5% BSA, the cells were incubated with the following primary antibodies: anti-*α*-smooth muscle actin (SMA, Abcam, ab5831), anti-vimentin (Abcam, ab92547), anti-CD31 (Dako, M0823), anti-vascular endothelial growth factor receptor 2 (VEGFR-2, Abcam, ab10972), anti-p53 (Abcam, ab131442), or anti-JMY (Abcam, ab217953) overnight at 4°C. The cells were incubated for 30 mins with the appropriate secondary antibody conjugated to fluorescein isothiocyanate (FITC). The nuclei were stained with 4′,6-diamidino-2-phenylindole (DAPI, Sigma-Aldrich). Briefly, the cells were washed twice with PBS and incubated for 5 min in the dark with 1 : 750 solution of stock DAPI (2 mg/mL). After rinsing with PBS, the slides were allowed to air dry at room temperature, and the cells were observed under an Afv10i confocal microscope (Olympus, Japan).

### 2.8. Western Blotting and Tube-Like Structure Formation Assay

Cells and tissues were collected and snap-frozen in liquid nitrogen using the ultrasonic cell-break method for five sec on ice. After centrifugation at 12,000 g for 30 min, the supernatant was collected. Protein lysates (30 *μ*g) were loaded onto SDS-PAGE gels and transferred to PVDF membranes. The PVDF membranes were blocked with 5% BSA and incubated overnight with monoclonal antibodies against *α*-SMA (1 : 1,000, Santa Cruz, CA, USA), vimentin (1 : 1,000), CD31 (1 : 2,000), VEGFR-2 (1 : 2,500), p53 (1 : 1,500), JMY (1 : 3,000), collagen-1/3 (1 : 2000), or Timp-1-4 (1 : 3000). The membranes were probed with a secondary antibody (1 : 4,000; Abcam). Immunoreactivity was determined via enhanced chemiluminescence (Millipore, USA). Glyceraldehyde-3-phosphate dehydrogenase (GAPDH, 1 : 10,000) was utilized as a control.

For detection of tube-like structure formation, Matrigel (300 *μ*L) was plated into the bottom of 6-well plates at 37°C for 30 min, and 10,000 CFs were seeded on the Matrigel and treated with exosomes containing miRNAs for 24 h. An inverted microscope (Olympus) was used to assess tube formation.

### 2.9. Animal Experimental Model

Three-month-old healthy male SD (Sprague-Dawley) rats (230-250 g) were used for experimentation in accordance with the “Guide for the Care and Use of Laboratory Animals” (National Institutes of Health publication 8th Edition, 2011). The protocol was approved by the Committee for the Ethics of Animal Experiments of the First Affiliated Hospital of Sun Yat-sen University. The rats were fixed on a board and anaesthetized via intraperitoneal administration of 100 mg/kg ketamine + 10 mg/kg xylazine. Surgery was performed under sterile conditions. We disinfected the chest of the rats and lightly pressed on the right side of the thorax, extruding the heart and inserting a needle 2 mm below the root of the left atrial appendage to a needle depth of 0.5 mm. We threaded the surface of the myocardium near the pulmonary arterial cone with a 6/0 suture, rapidly ligated the left anterior descending artery, closed the thorax, and finally sutured the incisions layer by layer. The rats were allowed to move freely after surgery and were injected with 400,000 U penicillin to prevent infection. Postoperative medication (subcutaneous injection of 0.05 mg/kg buprenorphine) at 12 h and 24 h after surgery was also used to alleviate any discomfort.

After the operation, when the animals recovered spontaneous breathing, rats whose electrocardiogram (ECG) showed significantly increased J points and/or tall and biphasic or inverted T waves and/or Q waves were considered to be MI rats. The animals that met the above ECG requirements were randomly divided into three groups: MI group (model), MI with exosomes containing miR-218-5p mimic group, and MI with exosomes containing miR-363-3p mimic group, with ten rats in each group. Pooled exosomes (300 *μ*g) derived from EPCs with miR-218-5p mimic or miR-363-3p mimic transfection were first resuspended in 150 *μ*L of PBS [36, 37] and injected intramyocardially with an insulin syringe through a 21-gauge needle into the border of the visually recognizable ischemic area at 24 h after surgery. The sham-operated group without any surgery and the control model rats were injected with 150 *μ*L of PBS. After eight weeks, echocardiography was used to detect cardiac function in the rats. Capillary density was observed and calculated under an optical microscope.

### 2.10. Histological Evaluation

The heart tissues of rats in the different groups were immediately dissected, fixed in 10% buffered formalin, and processed in a paraffin tissue processing machine. Seven-micrometer sections were stained with hematoxylin and eosin (H&E, G1005, Servicebio, China), Masson's trichrome (G1006, Servicebio, China), and Van Gieson (G1046, Servicebio, China), and the tissues were assessed. Representative photomicrographs were observed under a microscope. For measurement of infarct volumes, the excised left ventricles of the hearts were frozen and sectioned from the apex to the base into 3 mm slices. The slices were immersed in 2% TTC (2,3,5-triphenyltetrazolium chloride, T8877, Sigma-Aldrich) solution in PBS for 30 min at 37°C in the dark. The slices were fixed in 10% formaldehyde, and the area of the ischemic damage was measured using a morphometric program (Digi Cell 4.0).

### 2.11. Immunohistochemistry

Heart tissue slices were blocked by incubation with 3% H_2_O_2_ after deparaffinization and rehydration and washed with 0.05 M ethylenediaminetetraacetic acid (EDTA) followed by 4% paraformaldehyde. The tissues were incubated in 5% dry milk and 0.5% goat serum for 20 min. Then, 5 *μ*m sections were incubated with anti-von Willebrand factor (anti-vWF) antibody in the presence of 10% rabbit serum overnight at room temperature. The sections were incubated with horseradish peroxidase- (HRP-) conjugated goat anti-rabbit IgG secondary antibody for another two hours. Diaminobenzidine (DAB) staining (K5007, Dako) was used for examination. The slides were counterstained with Harris hematoxylin to stain cell nuclei. The heart tissue slices were also incubated with anti-*α*-SMA antibody and the respective secondary antibody. The slides were counterstained with DAPI to stain cell nuclei.

### 2.12. Statistical Analysis

All the data are presented as the means ± standard deviation (SD). Each *in vitro* experiment was performed in triplicate. All the data produced by qRT-PCR were based on at least 3 biological repeats with at least 3 technical repeats. Statistical significance between/among different treatment groups was calculated using one-way analysis of variance (ANOVA) followed by Dunnett's multiple comparison test or an unpaired Student *t*-test, as appropriate, using SPSS 19.0 software (SPSS Inc., Chicago, IL, USA). *P* < 0.05 was considered to be statistically significant.

## 3. Results

### 3.1. Overview of Small RNA EPC and EPC-Exos Sequencing Data

A previous study demonstrated that EPC-Exos play a vital role in CF proliferation and angiogenesis [18]. To investigate similarities or differences in specific patterns of miRNA expression between cells and exosomes, we first isolated exosomes and identified them via transmission electron microscopy (Supplemental Figure 1A), particle diameter size distribution (Supplemental Figure 1B and Source data), and western blot analysis (Supplemental Figure 1C). The distribution of exosomes in EPCs was assessed by PKH67 staining (Supplemental Figure 1D). In an initial effort to identify differentially expressed miRNAs in exosomes that could account for their functions relative to EPCs, we profiled the expression of miRNAs using DESeq2. A total of 7,342,859 and 8,898,449 clean reads were obtained with 6,997,238 and 8,813,698 adapter-trimmed reads (length ≥ 15 nucleotides) from EPCs and exosomes, respectively (Supplemental Table 5). Further analysis showed that the number of reads aligned to known human pre-miRNAs in miRbase21 was 4,691,984 and 127,888 in EPCs and exosomes, respectively (Supplemental Table 5. Unique small RNAs with lengths ranging from 16 to 30 nucleotides were obtained by filtering the low-quality reads or sequence reads with a length less than 15 nucleotides, and the length distribution of unique small RNA sequences (17–30 nucleotides) in the cells and exosomes was similar (Supplemental Figure 2A). The lengths of unique small RNAs in the cells and exosomes ranged between 20 and 24 nucleotides, with the most abundant size class being 22 nucleotides followed by 23 and 21 nucleotides (Supplemental Figure 2A). The new miRNAs were discovered using miRDeep2 [38]. Reads with a removed adaptor, sequence length ≥ 17 base pairs, and mismatch number ≤ 1 were used to perform novel miRNA prediction, and 144 novel miRNA candidates were detected in chromosomes of both cell types and exosomes (Supplemental Figure 2B and Supplemental Table 6).

### 3.2. Differential miRNA Expression Profile between EPCs and EPC-Exos

**Figure 1:**
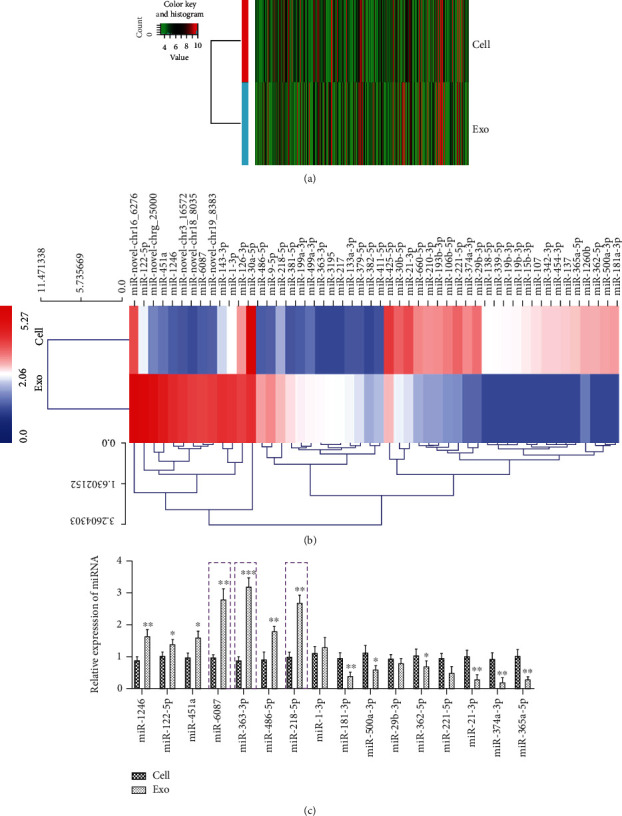
Differential miRNA expression profiles between EPCs and EPC-Exos. (a) Hierarchical clustering of differentially expressed miRNAs between EPCs and EPC-Exos, which are displayed on a scale from green (low) to red (high). (b) Hierarchical clustering heat map of the clearly distinct differentially expressed miRNAs in EPCs and EPC-Exos, ranging from red (relatively high miRNA expression) to blue (relatively low miRNA expression). The miRNAs in the heat map are clustered based on the relative expression patterns. (c) The relative expression levels of sixteen differentially expressed miRNAs (eight upregulated and eight downregulated) in EPC-Exos compared with EPCs were measured with qRT-PCR. The data are presented as the mean ± SD (*n* = 3). ^∗^*P* < 0.05, ^∗∗^*P* < 0.01, and ^∗∗∗^*P* < 0.001, EPC-Exos versus EPCs.

In total, 385 differentially expressed miRNAs were found in EPC-Exos compared with EPCs. Among these miRNAs, 100 were upregulated, and 285 were downregulated. A heat map of the hierarchical clustering of differential expression between EPCs and EPC-Exos was generated with a cut-off log2 (fold change) > 1 and *P* < 0.05 (Figure 1(a)). The top 25 upregulated miRNAs and top 25 downregulated miRNAs in the exosome group compared with EPCs are listed in Figure 1(b). Moreover, in an attempt to validate the reliability and robustness of the miRNA sequencing results, 16 differentially expressed miRNAs (eight upregulated: miR-1246, miR-122-5p, miR-451a, miR-6087, miR-363-3p, miR-486-5p, miR-218-5p, and miR-1-3p and eight downregulated: miR-181-3p, miR-500a-3p, miR-362-5p, miR-21-3p, miR-374a-3p, and miR-365a-5p in EPC-Exos) were selected and analyzed via qRT-PCR. The qRT-PCR results showed that miR-1246, miR-122-5p, miR-451a, miR-6087, miR-363-3p, miR-486-5p, and miR-218-5p were upregulated while miR-181-3p, miR-500a-3p, miR-362-5p, miR-21-3p, miR-374a-3p, and miR-365a-5p were downregulated in EPC-Exos compared with EPCs (Figure 1(c)). Furthermore, KEGG pathway analysis showed that upregulated miRNAs were significantly enriched in *Salmonella* infection, the HIF-1 signaling pathway, and cancer pathways, while downregulated miRNAs were significantly enriched in protein digestion and absorption, focal adhesions, and cancer pathways (Supplemental Figure 3A). Moreover, Gene Ontology (GO) analysis was performed to identify the key genes targeted by specific miRNAs. Upregulated miRNAs mainly participated in ion transport, regulation of anoikis, and neuromuscular functions (Supplemental Figure 3B), while downregulated miRNAs mainly participated in collagen and multicellular organism catabolic processes (Supplemental Figure 3C) for biological process (BP), cellular component (CC), and molecular function (MF).

### 3.3. miR-218-5p and miR-363-3p Promote CF Angiogenesis and Proliferation

**Figure 2:**
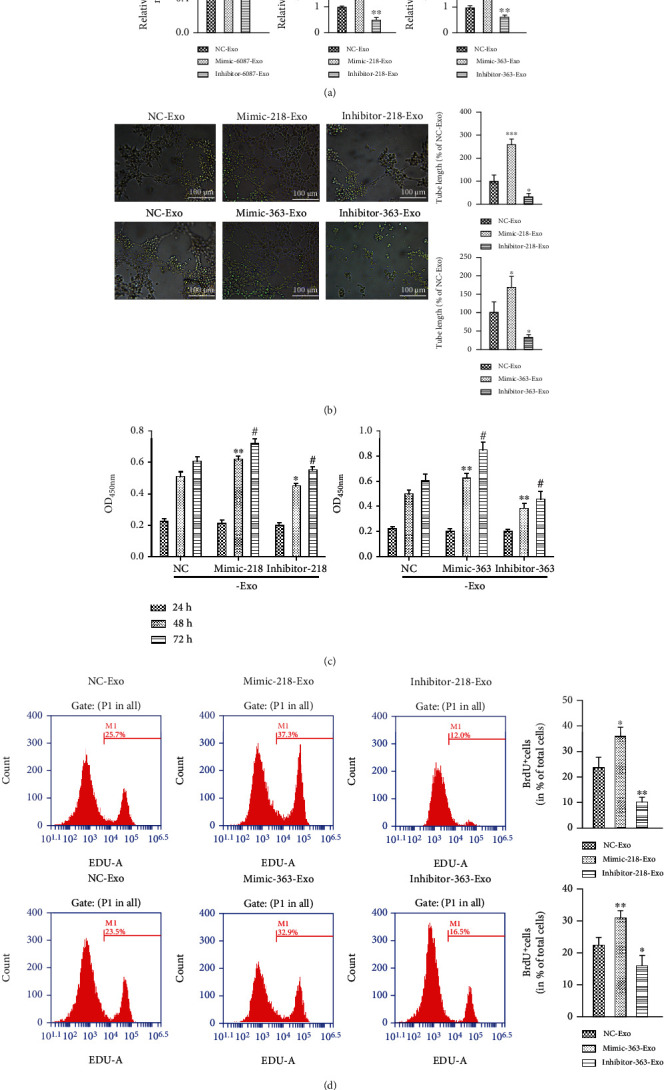
miR-218-5p and miR-363-3p promoted angiogenesis and proliferation of CFs. (a) The relative expression levels of miR-6087, miR-218-5p, and miR-363-3p mimics and inhibitors were measured with qRT-PCR. The data are presented as the mean ± SD (*n* = 3). ^∗∗^*P* < 0.01 and ^∗∗∗^*P* < 0.001, mimic-Exo or inhibitor-Exo versus NC-Exo. (b) Tube formation capability and relative tube length were increased in the miR-218-5p and miR-363-3p mimic-Exo groups and decreased in inhibitor-Exo groups. The data are presented as the mean ± SD (*n* = 3). ^∗^*P* < 0.05 and ^∗∗∗^*P* < 0.001, mimic-Exo or inhibitor-Exo versus NC-Exo. (c) CCK8 assay to detect the effect of exosomal miR-218-5p and miR-363-3p on cell proliferation. The data are presented as the mean ± SD (*n* = 3). ^∗#^*P* < 0.05 and ^∗∗^*P* < 0.01, mimic-Exo or inhibitor-Exo versus NC-Exo. (d) BrdU labelling and flow cytometry assay to detect the effect of exosomal miR-218-5p and miR-363-3p on cell proliferation. The data are presented as the mean ± SD (*n* = 3). ^∗^*P* < 0.05 and ^∗∗^*P* < 0.01, mimic-Exo or inhibitor-Exo versus NC-Exo.

To investigate the functional potential of miRNAs in MF, three miRNAs, namely, miR-6087, miR-218-5p, and miR-363-3p, which showed the most significant changes in the expression profiles, were selected for further examination. EPCs were transfected with mimics and inhibitors of the miRNAs, and their expression levels were detected in EPC-Exos using qRT-PCR (Figure 2(a)). The expression of miR-218-5p and miR-363-3p was significantly upregulated with miRNA mimic transfection and downregulated with miRNA inhibitor transfection in EPC-Exos compared with the NC-Exo group (Figure 2(a)). However, the expression of miR-6087 was not affected (Figure 2(a)). To determine whether the expression changes in miR-218-5p and miR-363-3p were caused by the number of exosomes secreted from EPCs, GW4869 (an inhibitor of neutral sphingomyelinase 2) was used to treat EPCs. As shown in Supplemental Figure 4A and 4B, the concentration of exosomes in EPCs treated with GW4869 was significantly decreased compared with the control. Further analysis showed that the expression of miR-218-5p and miR-363-3p was significantly increased in cells treated with GW4869 compared with control cells but significantly decreased in EPC-Exos (Supplemental Figure 4C and 4D). Therefore, miR-218-5p and miR-363-3p were selected for further experiments.

To investigate whether the two candidate miRNAs are involved in the mediation of CF proliferation and angiogenesis, CFs (NC-Exo), CFs+EPC-Exos with miR-218-5p mimic (mimic-218-Exo), CFs+EPC-Exos with miR-218-5p inhibitor (inhibitor-218-Exo), CFs+EPC-Exos with miR-363-3p mimic (mimic-363-Exo), and CFs+EPC-Exos with miR-363-3p inhibitor (inhibitor-363-Exo) were established. The number of capillary-like structures was increased in the mimic-218-Exo and mimic-363-Exo groups compared with the NC-Exo group and was decreased in the inhibitor-218-Exo and inhibitor-363-Exo groups (Figure 2(b)). Further analysis showed that the tube length in CFs with miRNA mimic-Exo transfection was significantly longer than that in the control, while shorter tube lengths were observed in the miRNA inhibitor-Exo groups (Figure 2(b)). These results indicate that miR-218-5p and miR-363-3p can promote angiogenesis of CFs. CCK8 assays demonstrated that treatment with miR-218-5p and miR-363-3p mimic-Exo dramatically increased cell viability compared to the control, while miRNA inhibitor-Exo significantly decreased cell viability at 48 h and 72 h (Figure 2(c)). These findings suggest that miR-218-5p and miR-363-3p can promote CF proliferation. To verify the results of this experiment, we performed a BrdU labelling assay. The results confirmed that EPC-Exos with miRNA mimic significantly promoted the proliferation of CFs, because the number of BrdU-positive cells found in CFs treated with mimics was greater than that in the inhibitor and control groups (Figure 2(d)). Therefore, miR-218-5p and miR-363-3p promoted CF proliferation.

In order to further confirm that miR-218-5p and miR-363-3p play important roles in CF proliferation and angiogenesis related to EPC-Exos, the function analysis of EPC-Exos to CF proliferation and angiogenesis was performed in a coculture system. CCK8 assays showed that cell viability was dramatically increased in the Exo group compared with control while significantly decreased when treated with GW4869 compared with the Exo group at 48 h (Supplemental Figure 5A). Further analysis showed that the number of capillary-like structures and the tube length in CFs was increased in the Exo group compared with control while decreased when treated with GW4869 compared with the Exo group (Supplemental Figure 5B). These results demonstrated that miR-218-5p and miR-363-3p which promoted CF proliferation and angiogenesis were from EPC-Exos.

To further confirm the changes in CF angiogenesis and proliferation caused by miR-218-5p and miR-363-3p, CFs were directly transfected with miR-218-5p and miR-363-3p. miR-218-5p and miR-363-3p were successfully overexpressed or suppressed in CFs harboring mimic or inhibitor, respectively (Supplemental Figure 6A). Further analysis showed that the expression of p53 was significantly upregulated in the mimic-218 (miR-218-5p mimic) group and downregulated in the inhibitor-218 (miR-218-5p inhibitor) group compared with the NC group. In addition, the expression of JMY was significantly downregulated in the mimic-363 (miR-363-3p mimic) group but upregulated in the inhibitor-363 (miR-363-3p inhibitor) group compared with the NC group (Supplemental Figure 6B). In addition, the results showed that tube length was significantly elevated in both the mimic-218 and mimic-363 groups and decreased in the inhibitor group compared with the NC group (Supplemental Figure 6C and 6D). BrdU labelling showed that the number of BrdU-positive cells found in CFs treated with mimic-218 or mimic-363 was more than that in the control groups, while the number in the inhibitor-treated group was decreased (Supplemental Figure 6E and 6F).

### 3.4. miR-218-5p and miR-363-3p Promoted Mesenchymal-Endothelial Transition in CFs

**Figure 3:**
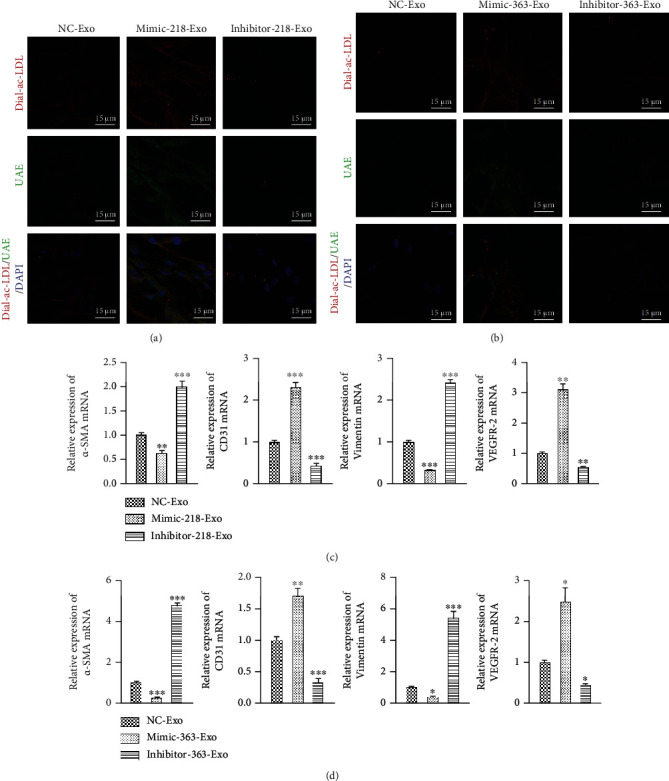
miR-218-5p and miR-363-3p promoted mesenchymal-endothelial transition in CFs. (a) A phagocytosis assay was performed to detect the engulfment of Dil-Ac-LDL and binding to endothelial-specific lectin FITC-UEA-1 by CFs treated with mimic-218-Exo or inhibitor-218-Exo. Magnification: 120x. (b) A phagocytosis assay was performed to detect the engulfment of Dil-Ac-LDL and binding to endothelial-specific lectin FITC-UEA-1 by CFs treated with mimic-363-Exo or inhibitor-363-Exo. Magnification: 120x. (c) qRT-PCR detection of the relative expression levels of CD31, VEGFR-2, *α*-SMA, and vimentin in CFs treated with mimic-218-Exo or inhibitor-218-Exo. The data are presented as the mean ± SD (*n* = 3). ^∗∗^*P* < 0.01 and ^∗∗∗^*P* < 0.001, mimic-Exo or inhibitor-Exo versus NC-Exo. (d) qRT-PCR detection of the relative expression levels of CD31, VEGFR-2, *α*-SMA, and vimentin in CFs treated with mimic-363-Exo or inhibitor-363-Exo. The data are presented as the mean ± SD (*n* = 3). ^∗^*P* < 0.05, ^∗∗^*P* < 0.01, and ^∗∗∗^*P* < 0.001, mimic-Exo or inhibitor-Exo versus NC-Exo.

**Figure 4:**
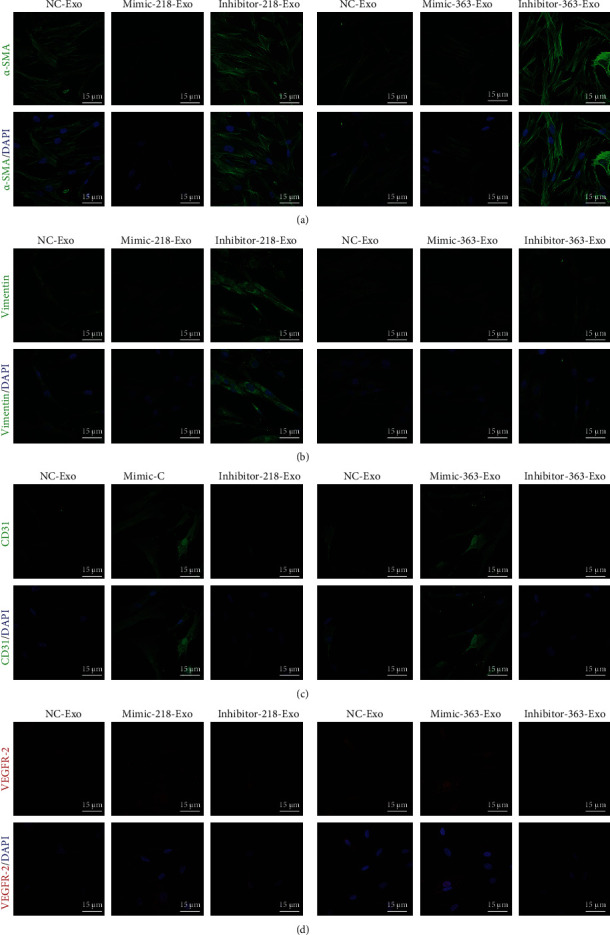
Immunofluorescence staining to detect the relative expression levels of *α*-SMA (a), vimentin (b), CD31 (c), and VEGFR-2 (d) in CFs treated with miR-218-5p and miR-363-3p mimics and inhibitors. The photographs were taken with a laser confocal microscope (Zeiss, LSM700), magnification: 120x.

**Figure 5:**
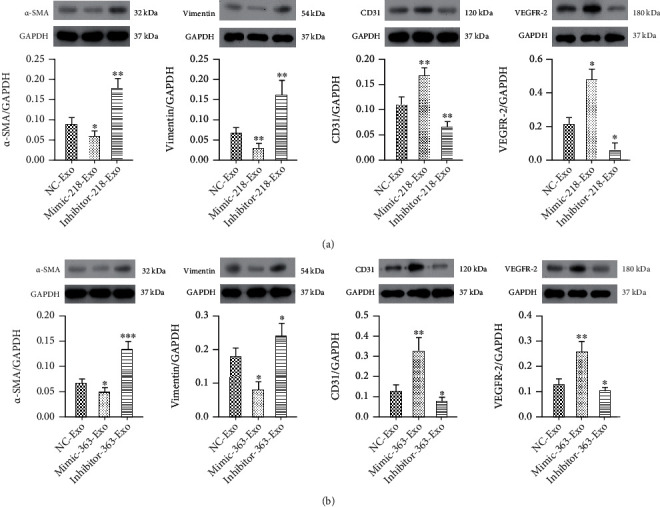
Western blot analysis to detect the relative expression levels of CD31, VEGFR-2, *α*-SMA, and vimentin in CFs treated with miR-218-5p (a) and miR-363-3p (b) mimic-Exo and inhibitor-Exo. The data are presented as the mean ± SD (*n* = 3). ^∗^*P* < 0.05, ^∗∗^*P* < 0.01, and ^∗∗∗^*P* < 0.001, mimic-Exo or inhibitor-Exo versus NC-Exo.

In our previous study, we demonstrated that EPC-Exos promoted MEndT to reduce MF [18]. To test the functional potential of miR-218-5p and miR-363-3p in MEndT, we detected the specific endothelial lineage characteristics of CFs using a phagocytosis assay. The results showed that phagocytosis in CFs dramatically increased in the mimic-218-Exo group (Figure 3(a)) and the mimic-363-Exo group (Figure 3(b)) in comparison with the NC-Exo group and the inhibitor-Exo group. We also assessed the expression of the endothelial cell markers cluster of differentiation 31 (CD31) and vascular endothelial growth factor receptor 2 (VEGFR-2) and the mesenchymal cell markers alpha-smooth muscle actin (*α*-SMA) and vimentin. qRT-PCR analysis showed that the expression of *α*-SMA and vimentin was significantly downregulated in the mimic-218-Exo group (Figure 3(c)) and the mimic-363-Exo group (Figure 3(d)), while the expression levels of CD31 and VEGFR-2 were upregulated compared to the NC-Exo group and the inhibitor-Exo group (Figures 3(c) and 3(d)). Immunofluorescence staining (Figures 4(a)–4(d)) and western blot analysis (Figures 5(a) and 5(b)) showed a decrease in the fibrosis markers *α*-SMA and vimentin and an increase in the endothelial markers CD31 and VEGFR-2 in the mimic-218-Exo and mimic-363-Exo groups. However, the opposite effects on the expression of these proteins were observed in the inhibitor-Exo groups. Transfection with miR-218-5p and miR-363-3p mimics induced MEndT in CFs, thereby playing an important role in ameliorating MF.

### 3.5. p53/JMY Were Respective Targets of miR-218-5p and miR-363-3p

**Figure 6:**
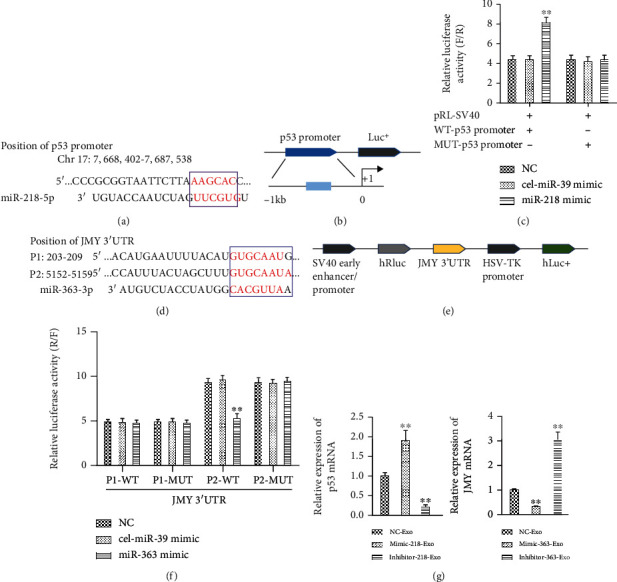
p53/JMY as a target of miR-218-5p and miR-363-3p. (a) The predicted binding sites for miR-218-5p in the p53 promoter. (b) Schematic diagram of pGL3-Basic vector construction harboring p53 promoter binding sites. (c) Relative luciferase activity (F/R) assay for miR-218-5p binding to the p53 promoter. cel-miR-39 mimic served as the negative control. The data are presented as the mean ± SD (*n* = 3), ^∗∗^*P* < 0.01, miR-218-5p mimic versus NC or cel-miR-39 mimic. (d) The predicted binding sites for miR-363-3p in the 3′UTR of JMY. (e) Schematic diagram of psi-Check2 vector construct harboring the 3′UTR of JMY binding sites. (f) Relative luciferase activity (R/F) assay for miR-363-3p in the 3′UTR of JMY. The data are presented as the mean ± SD (*n* = 3), ^∗∗^*P* < 0.01, miR-363-3p mimic versus NC or cel-miR-39 mimic. cel-miR-39 mimic served as the negative control. (g) qRT-PCR to detect the relative expression levels of p53 and JMY in CFs in the mimic-Exo and inhibitor-Exo groups. The data are presented as the mean ± SD (*n* = 3). ^∗^*P* < 0.05 and ^∗∗^*P* < 0.01, mimic-Exo or inhibitor-Exo versus NC-Exo.

To investigate the molecular mechanism of the miR-218-5p- and miR-363-3p-mediated effects on MF, we utilized TargetScan to identify the potential binding sites of miR-218-5p and miR-363-3p. Systematic bioinformatics analysis revealed that miR-218-5p could bind with the 5′-AAGCAC-3′ site in the promoter region of p53 and that miR-363-3p could bind with the 5′-GUGCAAU-3′ site in the 3′UTR of JMY mRNA at two sites (Figures 6(a) and 6(d)). Therefore, a luciferase reporter assay was used to identify interactions between miR-218-5p and the p53 promoter, and miR-363-3p and the 3′UTR of JMY. No obvious changes were observed in the NC and cel-miR-39 mimic groups, which harbored the wild-type and mutant p53 promoter. However, luciferase activity was significantly upregulated after cotransfection with miR-218-5p mimic and the reporter vector with the wild-type p53 promoter, while no significant change was observed with the mutant p53 promoter (Figures 6(b) and 6(c)). In addition, no obvious changes were found in the NC and cel-miR-39 mimic groups, harboring the wild-type and mutant JMY 3′UTR, regardless of whether the P1 or P2 binding site was present. However, luciferase activity was significantly decreased after cotransfection with miR-363-3p mimic and the reporter vector with wild-type JMY at the P2 binding site, while no significant change was observed for the P1 binding site (Figures 6(e) and 6(f)). Therefore, miR-218-5p could bind the p53 promoter region, and miR-363-3p could bind P2 sites in the 3′UTR of JMY.

**Figure 7:**
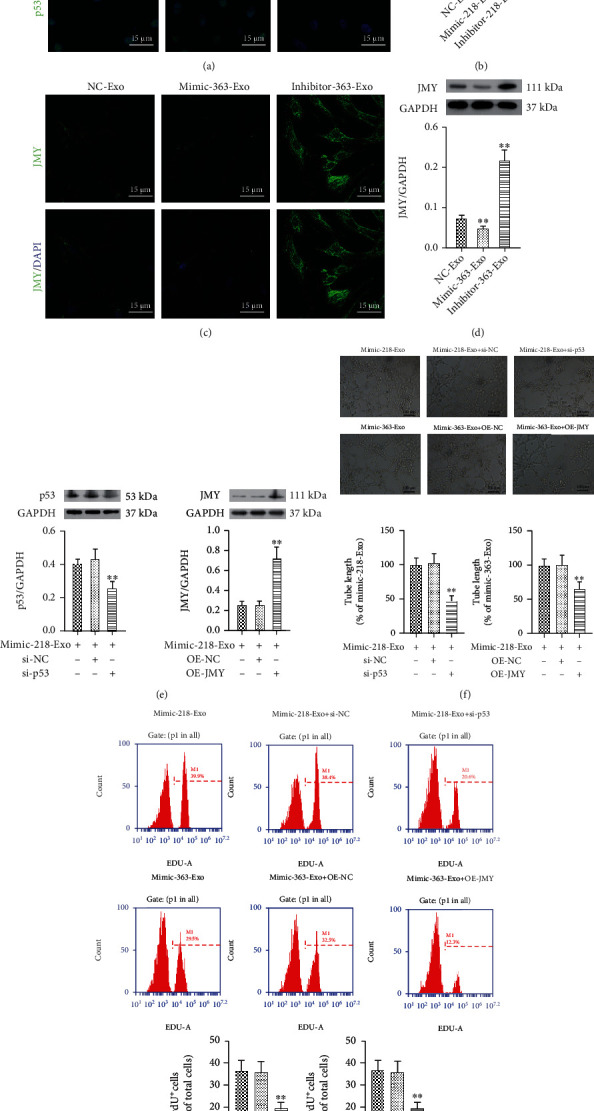
Effect of miR-218-5p and miR-363-3p on p53/JMY. (a, c) Immunofluorescence staining for the relative expression levels of p53 and JMY in CFs, in the miR-218-5p/miR-363-3p mimic-Exo and inhibitor-Exo groups. (b, d) Western blot analysis to detect the relative expression levels of p53 and JMY in miR-218-5p/miR-363-3p mimic-Exo and inhibitor-Exo groups in CFs, respectively. The photographs were taken with a laser confocal microscope (Zeiss, LSM700), magnification: 120x. The data are presented as the mean ± SD (*n* = 3). ^∗^*P* < 0.05 and ^∗∗^*P* < 0.01, mimic-Exo or inhibitor-Exo versus NC-Exo. (e) Western blot analysis to detect the relative expression levels of p53 and JMY in si-p53 and OE-JMY CF groups, respectively. The data are presented as the mean ± SD (*n* = 3). ^∗∗^*P* < 0.01, si-p53 or OE-JMY group versus mimic-218-Exo or mimic-363-Exo group. (f) The tube formation capability and relative tube length were decreased in the si-p53 and OE-JMY groups. The data are presented as the mean ± SD (*n* = 3). ^∗∗^*P* < 0.01, si-p53 or OE-JMY group versus mimic-218-Exo or mimic-363-Exo group. (g) BrdU labelling and flow cytometry assay to detect the effect of si-p53 and OE-JMY on cell proliferation. The data are presented as the mean ± SD (*n* = 3). ^∗∗^*P* < 0.01, si-p53 or OE-JMY group versus mimic-218-Exo or mimic-363-Exo group.

Furthermore, the mRNA expression of *TP53 (P53)* was upregulated in the mimic-218-Exo group and downregulated in the inhibitor-Exo group compared to the NC-Exo group (Figure 6(g)). Moreover, the mRNA expression of JMY was significantly downregulated in the mimic-363-Exo group and upregulated in the inhibitor-Exo group compared with the NC-Exo group (Figure 6(g)). The expression changes in TP53 and JMY were further verified by immunofluorescence staining (Figures 7(a) and 7(c)) and western blot analysis (Figures 7(b) and 7(d)). In addition, the expression of p53 was suppressed and JMY was overexpressed in CFs (Figure 7(e)) compared with the mimic-218-Exo and mimic-363-Exo groups, respectively. We also found that both tube length (Figure 7(f)) and the number of BrdU-positive cells (Figure 7(g)) were dramatically decreased in both the si-p53 and OE-JMY groups compared with the mimic-218-Exo and mimic-363-Exo groups, respectively. In general, our results indicate that EPC-Exos regulate the proliferation, angiogenesis, and tube formation of CFs through miR-218-5p- and miR-363-3p-mediated p53/JMY signaling pathways.

### 3.6. miR-218-5p and miR-363-3p Mimic Alleviated LCA Ligation-Induced MI

**Figure 8:**
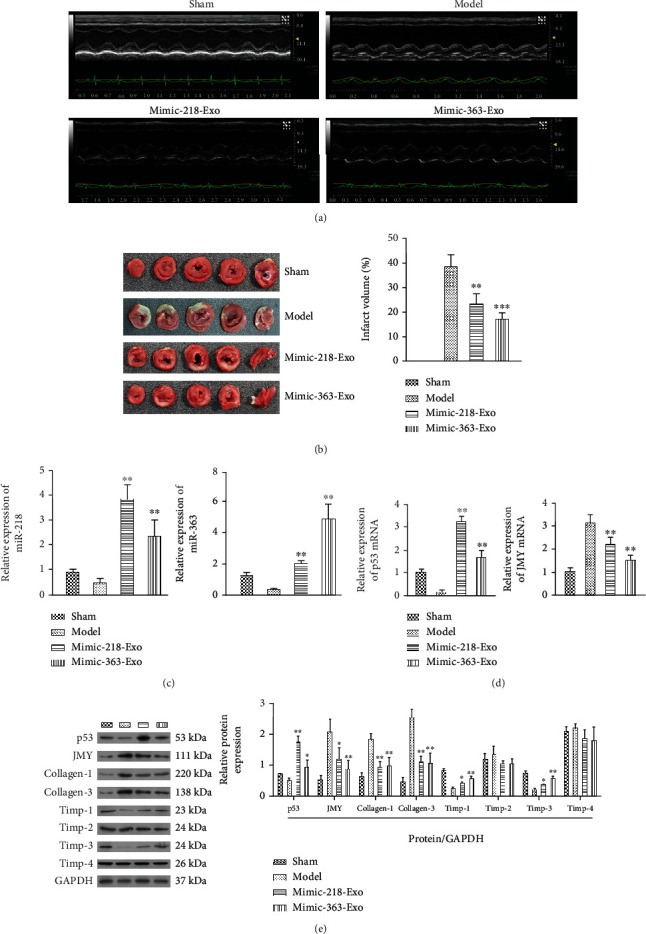
Effect of exosomal miR-218-5p and miR-363-3p on cardiac features and function in MI rats. (a) A representative ECG of LCA-induced MI rats in the different groups (sham, model, miR-218-5p, and miR-363-3p mimic-Exo). (b) TTC staining of heart tissues from the sham, model, and mimic-Exo groups. The data are presented as the mean ± SD (*n* = 10). ^∗∗^*P* < 0.01 and ^∗∗∗^*P* < 0.001, mimic-Exo versus model. (c, d) qRT-PCR to detect the relative expression levels of miR-218-5p, miR-363-3p, p53, and JMY in the mimic-Exo groups. (e) The protein expression of p53, JMY, collagen-1/3, and Timp-1-4 in the different groups. The data are presented as the mean ± SD (*n* = 10). ^∗^*P* < 0.05 and ^∗∗^*P* < 0.01, mimic-Exo versus model.

To investigate whether exosomal miR-218-5p and miR-363-3p can ameliorate LCA ligation-induced MI *in vivo*, we first constructed a MI rat model by injecting EPC-Exos containing miR-218-5p and miR-363-3p mimic. ECGs showed inverted QRS waves in the MI model group (model) compared to the sham-operated group (sham), suggesting successful establishment of an MI model (Figure 8(a)). Injection of EPC-Exos with miR-218-5p and miR-363-3p mimic attenuated the impaired cardiac function, indicating a therapeutic effect on MI (Figure 8(a)). Furthermore, LVEDd, LVESd, and LVIDs were increased in the model group, and these effects were partially reversed by treatment with EPC-Exos containing miR-218-5p and miR-363-3p mimic (Supplemental Table 4). Similarly, the decreased parameter values in the model rats, including HR, LVIDd, SV, LVEF, FS, CO, LVPWT, and LVAWT, were also partially restored to the values seen in sham rats after treatment with EPC-Exos containing miR-218-5p and miR-363-3p mimic (Supplemental Table 4). For TTC staining, a large core volume of tissue injury was found in the model group (Figure 8(b)). However, the core volume steadily decreased to approximately 50% in the mimic-218-Exo and mimic-363-Exo groups compared with the model group (Figure 8(b)). Subsequently, significant increased expression of miR-218 and miR-363 was observed in both the mimic-363-Exo and mimic-218-Exo groups compared with the sham and model groups (Figure 8(c)). Furthermore, we found that the expression of JMY was significantly decreased in the mimic-363-Exo group while the expression of p53 was significantly elevated in the mimic-218-Exo group compared with the model group (Figures 8(d) and 8(e)). Western blotting also confirmed these results (Figure 8(f)). In addition, we detected the expression of collagen-1, collagen-3, Timp-1, Timp-2, Timp-3, and Timp-4. As shown in Figure 8(f), the expression levels of collagen-1 and collagen-3 were significantly decreased, while those of Timp-1 and Timp-3 were upregulated (no change in the expression of Timp-2 and Timp-4) in both the mimic-218-Exo and mimic-363-Exo groups compared with the model group (Figure 8(f)). Therefore, exosomal miR-218-5p and miR-363-3p can reduce cardiac dysfunction in MI rats.

**Figure 9:**
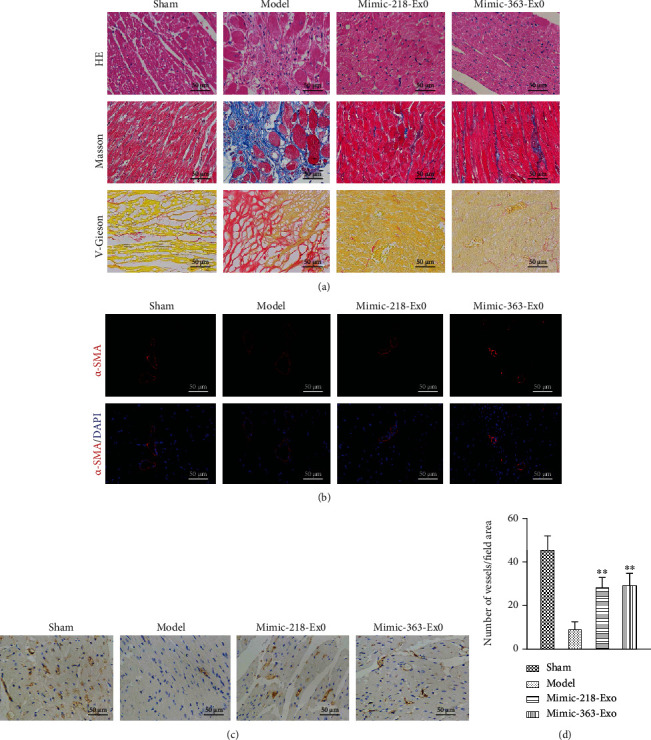
Exosomal miR-218-5p and miR-363-3p alleviate LCA-induced myocardial infarction. (a) H&E, Masson, and Van Gieson staining of heart tissues treated with EPC-Exos containing miR-218-5p mimic or miR-363-3p mimic. Magnification: 40x. (b) Immunofluorescence staining for the relative expression levels of *α*-SMA in heart tissues treated with EPC-Exos containing miR-218-5p mimic or miR-363-3p mimic. Magnification: 40x. (c) Immunohistochemistry for vWF in heart tissues treated with EPC-Exos containing miR-218-5p mimic or miR-363-3p mimic. (d) The number of vessels/field area in the remodeled left ventricle. The data are presented as the mean ± SD (*n* = 3). ^∗∗^*P* < 0.01, mimic versus model.

Histological examination of the rat heart tissues was further performed. As shown in Figure 9(a), after the challenge of the LCA ligation, H&E staining of heart tissues showed typical MI pathological traits. However, these histopathological changes were clearly attenuated in the mimic-218-Exo and mimic-363-Exo groups, reflected by visible fibers with cross-linked-shaped striated muscle tissue containing nuclei located on the periphery of the cell and remodeling of the intercalated disc connections (Figure 9(a)). Masson and Van Gieson staining confirmed a reduced degree of cardiac fibrosis with downregulation of collagen expression in the mimic-218-Exo and mimic-363-Exo groups (Figure 9(a)). Moreover, the expression of *α*-SMA was dramatically reduced in the mimic-218-Exo and mimic-363-Exo groups compared to the model group (Figure 9(b)). Furthermore, immunohistochemistry assays showed that the von Willebrand factor (vWF) expression level was partially restored in the mimic-218-Exo and mimic-363-Exo groups compared to the model group (Figure 9(c)). As a risk factor for patients with MI, vWF plays a vital role in hemostasis and is always elevated in the MI rat model [39]. We also quantified capillary density. In line with the histological examinations, the number of vessels/field area in the remodeled left ventricle in the mimic-218-Exo and mimic-363-Exo groups was increased compared with that in the model group (Figure 9(d)). These findings confirm that exosomal miR-218-5p and miR-363-3p can alleviate LCA ligation-induced MI.

## 4. Discussion

Cardiovascular disease not only places a severe burden on affected patients and their families but also influences society and economic development, underscoring the need for innovative new therapies [40]. Myocardial infarction-induced remodeling of the left ventricle is the most common cause of CHF, which is the leading cause of cardiovascular mortality [1]. Therefore, exploring the regulation mechanisms underlying MI is of great significance for control of cardiovascular disease development. Accumulating evidence has shown that exosomes can play a role in therapeutic effects in MI and that miRNA plays an important role in MI [41–43]. However, the molecular mechanism of EPC-Exos miRNAs in MI is rarely studied. Here, we examined the role of exosome-derived miRNA in regulating angiogenesis, proliferation, and mesenchymal-endothelial transition of CFs during MI *in vitro* and *in vivo*. The results showed that exosomal miR-218-5p and miR-363-3p from endothelial progenitor cells promoted angiogenesis, proliferation, and mesenchymal-endothelial transition of CFs by targeting the p53 promoter and JMY 3′UTR, respectively, which further alleviated LCA-induced chronic myocardial infarction. Our study will shed new light on Exo-miRNA-based therapy for MI.

MicroRNAs (miRNAs) are small noncoding RNAs that block translation or induce degradation of mRNA and thereby control patterns of gene expression. Increasing studies have found that miRNAs play crucial roles in MI [44–46]. For example, microRNA-133 overexpression promotes the therapeutic efficacy of mesenchymal stem cells in acute myocardial infarction [47]; Li et al. demonstrated that intravenous miR-144 reduces left ventricular remodeling after myocardial infarction [48]. miR-208, miR-494, miR-499, and miR-1303 expression levels are considered to be biomarkers in the early diagnosis of acute myocardial infarction [49]. MicroRNA-21 was fund to mediate the protective effect of cardiomyocyte-derived conditioned medium on ameliorating myocardial infarction in rats [50]. These results demonstrate that different miRNAs play important roles in MI. As small nanometer-sized vesicles and intercellular shuttles, exosomes essentially transfer loading proteins and RNAs for offloading in target cells and modulate gene and protein expression, thereby regulating cell activity [42, 51]. And Luo et al. revealed that exosomes from miR-126-overexpressing ADSCS were therapeutic in relieving acute myocardial ischemic injury [52]. Tian et al. demonstrated that MI-induced microRNA-enriched exosomes contribute to cardiac Nrf2 dysregulation in chronic heart failure [30]. Zhu et al. found that hypoxia-elicited mesenchymal stem cell-derived exosomes facilitated cardiac repair through miR-125b-mediated prevention of cell death in myocardial infarction [53]. Recently, it showed that serum exosomal miR-21, miR-126, and PTEN are novel biomarkers for diagnosis of acute coronary syndrome [54]. These results demonstrate that exosomal miRNA plays key roles in MI and might provide a novel therapeutic pathway for relieving MI. In the present study, 385 differentially expressed miRNAs, with 100 upregulated and 285 downregulated, were found in EPC-Exos compared with EPCs by sequencing and profiling miRNA expression in EPC-Exos. miR-218-5p and miR-363-3p showed the most significant changes in the expression profiles, demonstrating the important roles of miR-218-5p and miR-363-3p in MI.

Several miRNAs have been shown to control important processes that contribute to the pathophysiological consequences of MI, such as regulating cardiomyocyte cell death and postischemic neovascularization, controlling cardiomyocyte proliferation, or interfering with cardioprotective effects mediated by stem or progenitor cells [55–57]. In addition, miRNAs can be used for direct reprogramming of cardiac fibroblasts into cardiomyocytes [41]. A recent study showed that EPC-Exos contribute to CF proliferation and angiogenesis and ameliorate MF [18]. Arif et al. revealed that microRNA-210-mediated proliferation, survival, and angiogenesis promoted cardiac repair postmyocardial infarction in rodents [58]. Cardiomyocyte-derived exosomal microRNA-92a mediated postischemic myofibroblast activation both *in vitro* and *ex vivo* [59]. In addition, Fan et al. demonstrated that microRNA-210 promotes angiogenesis in acute myocardial infarction [60]; M1-like macrophage-derived exosomes suppressed angiogenesis and exacerbate cardiac dysfunction in a myocardial infarction microenvironment [61]. Ma et al. revealed that microRNA-132 delivered by mesenchymal stem cell-derived exosomes can promote angiogenesis in MI [62]. Therefore, we speculated that miR-218-5p and miR-363-3p might be involved in CF proliferation and angiogenesis in MI. Consistent with this hypothesis, our study revealed that miR-218-5p and miR-363-3p mimics promoted angiogenesis and proliferation in CFs. In our previous study, we demonstrated that EPC-Exos promoted MEndT to reduce MF [18]. In addition, Bayoumi et al. demonstrated that microRNA-532 protects the heart in acute myocardial infarction via endothelial-to-mesenchymal transition by suppressing prss23 [63]. Our *in vivo* and *in vitro* experiments showed that miR-218-5p and miR-363-3p induced MEndT of CFs via upregulation of endothelial cell markers (CD31 and VEGFR-2) and downregulation of fibrosis markers (*α*-SMA and vimentin). These results demonstrate that miR-218-5p and miR-363-3p mimics promoted CF angiogenesis and proliferation by promoting mesenchymal-endothelial transition in CFs.

Increasing numbers of targets regulated by miRNAs have been reported in MI, such as PPAR-*γ*, Smad7, bak1, klf13, and TXNIP [64–66]. In the present study, we found that p53 and JMY were directly regulated by miR-218-5p and miR-363-3p. In addition, miR-218-5p was found to activate p53 expression, and these results are consistent with previous research [67]. p53 and JMY have been demonstrated to participate in angiogenesis and proliferation in many diseases, especially in cancer [68–70]. However, the function of p53 and JMY in MI is rarely studied. In the present study, we found that suppression of p53 and overexpression of JMY could attenuate the CF angiogenesis and proliferation affected by miR-218-5p and miR-363-3p. In addition, evidence from other groups has shown a switch regulation between p53 and MEndT in CFs, which in the absence of p53 reduces the formation of fibroblast-derived endothelial cells and leads to cardiac injury, while activation of p53 promotes MEndT, resulting in cardiac function improvement [71]. Interestingly, in addition to the involvement of transcription cofactors in the p53 signaling pathway, JMY decreases the expression of adhesion molecules in the cadherin family and contributes to assembly of the actin cytoskeleton and to nucleation of new filaments [72]. Cadherins are endothelial-specific markers, and thus, a decrease in cadherin expression reflects a decrease in endothelial-mesenchymal transition (EMT) [18]. Taken together, these results demonstrate that upregulation of p53 by miR-218-5p and downregulation of JMY by miR-363-3p might relieve MF by inducing MEndT.

In addition, we identified the effect of exosomal miR-218-5p and miR-363-3p in MI by constructing an LCA ligation-induced MI rat model. The results indicated that arrhythmia, LVEDd, LVESd, LVIDs, HR, LVIDd, SV, LVEF, FS, CO, LVPWT, and LVAWT were partially restored by injection of EPC-Exos containing miR-218-5p and miR-363-3p mimic. Further analysis showed that EPC-Exos containing miR-218-5p and miR-363-3p mimic effectively suppressed infarction-induced myocardial damage by inhibiting inflammation and macrophage infiltration, downregulating collagen expression, and increasing capillary density, as shown by histochemical staining and analysis of collagen-1, collagen-3, and Timp-1-4 expression. These results demonstrate that exosomal miR-218-5p and miR-363-3p can reduce the cardiac dysfunction in MI rats and might provide new targets for MI therapy. However, future animal studies are required to guidance administration of miR-218-5p and miR-363-3p mimic to treat MI in the clinical context. Furthermore, the specific synthesis mechanism and function of exosomes are not very detailed and there is a lack of cost-effective exosome separation technology. In addition, future studies need to classify whether miRNA packaging into exosomes and exosomal uptake is a selective/stimulus-dependent process because there is no evidence to suggest functional differences between exosomal miRNAs and free ones and whether exosomal and free miRNAs are differentially regulated in response to stimulation. However, although the current biology of exosomes is still immature, more and more interest and capital investment will accelerate the progress of basic research and clinical transformation of exosomes.

Several limitations should be addressed in this study. Firstly, the expression of miRNAs in the EPC-Exos has been detected by qRT-PCR, while the localization of these miRNAs in the EPC-Exos may still require future experiments for validation. Secondly, the present study only examined two miRNAs (miR-218-5p and miR-363-3p) based on the microarray data, and future studies should carry out to further explore the functional roles of other potential miRNAs based on the microarray screening. Thirdly, the MI animal model was the nonreperfused ischemic injury model, and future studies may investigate the role of EPC-Exos in the reperfused ischemic injury model.

## 5. Conclusions

Our study shows that EPC-Exos containing miR-218-5p and miR-363-3p mimic exerted a protective effect against MI-induced myocardial damage and MF. Our *in vitro* results indicate that miR-218-5p and miR-363-3p mimic have protective properties against MF by targeting p53/JMY-mediated cell apoptosis and MEndT. The present study provides novel insight into the molecular mechanism underlying the effect of EPC-Exos containing miR-218-5p and miR-363-3p mimic on attenuation of myocardial damage.

## Data Availability

The datasets supporting the conclusions of this article are included within the article and its additional files.

## Abbreviations

MI: Myocardial infarction
EPC-Exos: Endothelial progenitor cell-derived exosomes
MF: Myocardial fibrosis
CFs: Cardiac fibroblasts
miRNAs: MicroRNAs
qRT-PCR: Quantitative real-time polymerase chain reaction
JMY: Junction-mediating and regulatory protein
LCA: Left coronary artery
EPCs: Endothelial progenitor cells
CHF: Chronic heart failure
LV: Left ventricle
MEndT: Mesenchymal-endothelial transition
EMT: Endothelial-mesenchymal transition
mRNA: Messenger RNA
Nrf2: Nuclear factor (erythroid-derived 2)-like 2
GO: Gene Ontology
KEGG: Kyoto Encyclopedia of Genes and Genomes
Dil-Ac-LDL: Acetylated low-density lipoprotein, labelled with 1,1-dioctadecyl-3,3,3,3-tetramethylindocarbocyanine
CD31: Cluster of differentiation 31
BrdU: 5-Bromo-2′-deoxyuridine
CCK8: Cell Counting Kit-8
UAE-1: Ulex europaeus agglutinin I
PBS: Phosphate-buffered saline
EGM-2: Endothelial cell growth medium-2
DMEM: Dulbecco's modified Eagle's medium
FBS: Fetal bovine serum
*α*-SMA: *α*-Smooth muscle actin
VEGFR-2: Vascular endothelial growth factor receptor 2
FITC: Fluorescein isothiocyanate
DAPI: 4′,6-Diamidino-2-phenylindole
TBST: Tris-buffered saline-Tween-20
GAPDH: Glyceraldehyde-3-phosphate dehydrogenase
SD: Sprague-Dawley
ECG: Electrocardiogram
H&E: Hematoxylin and eosin
TTC: 2,3,5-Triphenyltetrazolium chloride
EDTA: Ethylenediaminetetraacetic acid
vWF: von Willebrand factor
HRP: Horseradish peroxidase
DAB: Diaminobenzidine
HR: Heart rate
bpm: Beat per minute
LVESd: Left ventricular end-systolic diameter
LVEDd: Left ventricular end-diastolic diameter
LVIDs: Left ventricular internal diameter end systole
LVIDd: Left ventricular internal diameter end diastole
SV: Stroke volume
LVEF: Left ventricular ejection fraction
FS: Fractional shortening
CO: Cardiac output
LVPWT: Left ventricular posterior wall thickness
LVAWT: Left ventricular anterior wall thickness.

## Supplementary Materials

Supplementary Materials Supplemental Figure 1: characterization of EPC-Exos. (A) Transmission electron microscopy analysis of EPC-Exos in sparse regions. Scale bar: 100 nm. (B) The particle diameter size distribution of EPC-Exos. (C) Western analysis of the surface proteins of exosomes (CD63, Alix, TSG101, and Calnexin). (D) PKH67 staining of EPC-Exos. Supplemental Figure 2: reads statistics for miRNA sequencing data in EPCs and EPC-Exos. (A) Length distribution of small RNAs in EPCs and EPC-Exos. (B) New miRNAs were discovered using miRDeep2. Supplemental Figure 3: Gene Ontology (GO) enrichment and KEGG pathway analysis of differentially expressed miRNAs in EPC-Exos vs EPCs. (A) KEGG pathway analysis of the upregulated differentially expressed miRNAs (left panel) and downregulated miRNAs (right panel) in EPC-Exos vs. EPCs. The *P* value (ease-score, Fisher's *P* value, or hypergeometric *P* value, cutoff at 0.05) denotes the significance of the pathway correlated with the conditions. The lower the *P* value is, the more significant the pathway is. (B) Gene Ontology (GO) enrichment analysis of upregulated differentially expressed miRNAs in EPC-Exos vs. EPCs. The top ten enrichment score counts in the GO biological process classification for biological process, cellular components, and molecular function are listed. (C) Gene Ontology (GO) enrichment analysis of downregulated differentially expressed miRNAs in EPC-Exos vs. EPCs. Supplemental Figure 4: characterization of EPC-Exos treated with GW4869. (A) Transmission electron microscopy analysis of EPC-Exos and EPC-Exos treated with GW4869 in sparse regions. Scale bar: 100 nm. (B) The particle diameter size distribution and concentration of EPC-Exos with and without GW4869 treatment. (C, D) The relative expression of miR-218-5p and miR-363-3p in EPCs and EPC-Exos with and without GW4869 treatment. ^∗∗^*P* < 0.01, GW4869 versus control. Supplemental Figure 5: function analysis of EPC-Exos to CF proliferation and angiogenesis treated with or without GW4869. (A) CCK8 assays to cell viability of CFs treated with EPC-Exo or GW4869-Exo. (B) Capillary-like structures and the tube length in CFs treated with EPC-Exo or GW4869-Exo. The data are presented as the mean ± SD (*n* = 3). Control group, without any treatment; Exo group, CFs coincubation with EPC-Exosomes; GW4869-Exo, CFs coincubation with EPC-Exosomes which treated with GW4869. ^∗∗^*P* < 0.01, Exo versus control; ^##^*P* < 0.01, GW4869-Exo versus Exo. Supplemental Figure 6: angiogenesis and proliferation of CFs treated with miR-218-5p and miR-363-3p directly. (A) The relative expression levels of miR-218-5p and miR-363-3p were measured with qRT-PCR in overexpressing and suppressing miR-218-5p and miR-363-3p groups, respectively. The data are presented as the mean ± SD (*n* = 3). ^∗^*P* < 0.05 and ^∗∗^*P* < 0.01, mimic or inhibitor versus NC. (B) The expression of p53 and JMY was detected by western blotting in overexpressing and suppressing miR-218-5p and miR-363-3p groups, respectively. (C, D) The tube formation capability and relative tube length were increased in the mimic-218 and mimic-363 groups. The data are presented as the mean ± SD (*n* = 3). ^∗∗^*P* < 0.01, mimic-218 or mimic-363 groups versus NC. (E, F) BrdU labelling and flow cytometry assay to detect the effect of miR-218-5p and miR-363-3p on cell proliferation. The data are presented as the mean ± SD (*n* = 3). ^∗^*P* < 0.05 and ^∗∗^*P* < 0.01, mimic or inhibitor group versus NC. Source data: particle size analysis of exosomes. Supplemental Table 1: list of primers for qRT-PCR to detect the 16 differentially expressed miRNAs. Supplemental Table 2: list of primers for construction of the wild-type and mutant p53 promoter and JMY 3′UTR used in the luciferase assays. Supplemental Table 3: list of miRNA mimic and inhibitor. Supplemental Table 4: effect of EPC-Exos containing miR-218-5p mimic or miR-363-3p mimic on the cardiac ultrasonography index of LCA ligation-induced myocardial infarction rats. Supplemental Table 5: reads statistics for cell and exosome samples. Supplemental Table 6: novel miRNAs predicted by miRDeep2.
