# Supplementary material for: Exosomal miR-218-5p/miR-363-3p from Endothelial Progenitor Cells Ameliorate Myocardial Infarction by Targeting the p53/JMY Signaling Pathway
Source: Oxid Med Cell Longev. 2021 Jul 16;2021:5529430. doi: 10.1155/2021/5529430 (PMC8302385; doi:10.1155/2021/5529430)
Supplement: Supplementary Materials — Supplemental Figure 1: characterization of EPC-Exos. (A) Transmission electron microscopy analysis of EPC-Exos in sparse regions. Scale bar: 100 nm. (B) The particle diameter size distribution of EPC-Exos. (C) Western analysis of the surface proteins of exosomes (CD63, Alix, TSG101, and Calnexin). (D) PKH67 staining of EPC-Exos. Supplemental Figure 2: reads statistics for miRNA sequencing data in EPCs and EPC-Exos. (A) Length distribution of small RNAs in EPCs and EPC-Exos. (B) New miRNAs were discovered using miRDeep2. Supplemental Figure 3: Gene Ontology (GO) enrichment and KEGG pathway analysis of differentially expressed miRNAs in EPC-Exos vs EPCs. (A) KEGG pathway analysis of the upregulated differentially expressed miRNAs (left panel) and downregulated miRNAs (right panel) in EPC-Exos vs. EPCs. The P value (ease-score, Fisher's P value, or hypergeometric P value, cutoff at 0.05) denotes the significance of the pathway correlated with the conditions. The lower the P value is, the more significant the pathway is. (B) Gene Ontology (GO) enrichment analysis of upregulated differentially expressed miRNAs in EPC-Exos vs. EPCs. The top ten enrichment score counts in the GO biological process classification for biological process, cellular components, and molecular function are listed. (C) Gene Ontology (GO) enrichment analysis of downregulated differentially expressed miRNAs in EPC-Exos vs. EPCs. Supplemental Figure 4: characterization of EPC-Exos treated with GW4869. (A) Transmission electron microscopy analysis of EPC-Exos and EPC-Exos treated with GW4869 in sparse regions. Scale bar: 100 nm. (B) The particle diameter size distribution and concentration of EPC-Exos with and without GW4869 treatment. (C, D) The relative expression of miR-218-5p and miR-363-3p in EPCs and EPC-Exos with and without GW4869 treatment. ∗∗P < 0.01, GW4869 versus control. Supplemental Figure 5: function analysis of EPC-Exos to CF proliferation and angiogenesis treated with or without GW486 [file 5529430.f1.zip › Source data for particle size analysis_R1_submission (1).docx]

| NTA Single Analysis Summary File | |
| --- | --- |
| Created with NTA 3.2 Dev Build 3.2.16 | |
|  |  |
| [Experiment Details] | |
| Software Version | NTA 3.2 Dev Build 3.2.16 |
| Experiment Name | Capture 16-51-38.nano |
| Sample Name | |
| Operator Name | XH 2 30X |
| Time Captured | 16:51:38 |
| Pre-treatment | |
| Diluent |  |
| Remarks |  |
|  |  |
| Filename: | Capture 16-51-45 |
|  |  |
| [Conditions] | |
| Temperature/C | 23.687 |
| Viscosity/cP | 0.915932 |
| Camera Type | sCMOS |
| Laser Type | Blue405 |
| Camera Level | 14 |
| Slider Shutter | 1259 |
| Slider Gain | 366 |
| Shutter/ms | 31.48 |
| Camera Histogram Upper Limit | 6588 |
| Camera Histogram Lower Limit | 0 |
| Frame rate/fps | 24.9825 |
| Syringe Pump Speed/AU | 0 |
|  |  |
| [Settings] | |
| Detection Threshold | 3 |
| Max Jump Mode | Auto |
| Max Jump Distance | 14.4348 |
| Blur | Auto |
| Min Track Length | Auto |
| First frame | 0 |
| Total frames analysed | 1498 |
|  |  |
| [Exclusion Regions] | |
| No Exclusion Regions Used | |
|  |  |
| [Results] |  |
| Dilution factor (concentrations adjusted for this factor) | |
| Concentration (Particles / ml) | 4.36E+06 |
| Particles per frame | 0.2 |
| Centres per frame | 0.3 |
| Completed tracks | 84 |
| X-Drift (pix/frame) | -1.4 |
| Y-Drift (pix/frame) | 0 |
|  |  |
| [Information] | |
| Completed Tracks | <100 |
| Concentration | Very low concentration |
| Video length | OK |
| Noise level | Noise detected |
| Vibration detected | No |
| Vibration correction applied | No |
| Settings changed? | No |
| Errors (1) |  |
| Errors (2) |  |
|  |  |
| [Data Included] | |
| Size distribution - Number weighting - With Percentiles | |
|  |  |
| [Size Data] | |
| Analysis Method | FTLA |
| Weighting | Number |
| Filename | Capture 16-51-45 |
| Mean | 65.3 |
| Mode | 64.7 |
| SD | 6.1 |
| D10 | 56.6 |
| D50 | 64.1 |
| D90 | 72.3 |
| Valid Tracks | 1 |
| Graph Data | |
| Bin centre (nm) | Concentration (particles / ml) |
| 0.5 | 0 |
| 1.5 | 0 |
| 2.5 | 0 |
| 3.5 | 0 |
| 4.5 | 0 |
| 5.5 | 0 |
| 6.5 | 0 |
| 7.5 | 0 |
| 8.5 | 0 |
| 9.5 | 0 |
| 10.5 | 0 |
| 11.5 | 0 |
| 12.5 | 0 |
| 13.5 | 0 |
| 14.5 | 0 |
| 15.5 | 0 |
| 16.5 | 0 |
| 17.5 | 0 |
| 18.5 | 0 |
| 19.5 | 0 |
| 20.5 | 0 |
| 21.5 | 0 |
| 22.5 | 0 |
| 23.5 | 0 |
| 24.5 | 0 |
| 25.5 | 0 |
| 26.5 | 0 |
| 27.5 | 0 |
| 28.5 | 0 |
| 29.5 | 0 |
| 30.5 | 0 |
| 31.5 | 0 |
| 32.5 | 0 |
| 33.5 | 0 |
| 34.5 | 0 |
| 35.5 | 0 |
| 36.5 | 0 |
| 37.5 | 0 |
| 38.5 | 0 |
| 39.5 | 2 |
| 40.5 | 6 |
| 41.5 | 18 |
| 42.5 | 46 |
| 43.5 | 111 |
| 44.5 | 253 |
| 45.5 | 545 |
| 46.5 | 1110 |
| 47.5 | 2145 |
| 48.5 | 3944 |
| 49.5 | 6911 |
| 50.5 | 11561 |
| 51.5 | 18499 |
| 52.5 | 28363 |
| 53.5 | 41735 |
| 54.5 | 59027 |
| 55.5 | 80362 |
| 56.5 | 105460 |
| 57.5 | 133574 |
| 58.5 | 163491 |
| 59.5 | 193602 |
| 60.5 | 222050 |
| 61.5 | 246931 |
| 62.5 | 266515 |
| 63.5 | 279449 |
| 64.5 | 284917 |
| 65.5 | 282711 |
| 66.5 | 273237 |
| 67.5 | 257425 |
| 68.5 | 236595 |
| 69.5 | 212286 |
| 70.5 | 186080 |
| 71.5 | 159451 |
| 72.5 | 133654 |
| 73.5 | 109655 |
| 74.5 | 88109 |
| 75.5 | 69375 |
| 76.5 | 53557 |
| 77.5 | 40558 |
| 78.5 | 30144 |
| 79.5 | 21999 |
| 80.5 | 15772 |
| 81.5 | 11113 |
| 82.5 | 7699 |
| 83.5 | 5247 |
| 84.5 | 3519 |
| 85.5 | 2323 |
| 86.5 | 1510 |
| 87.5 | 967 |
| 88.5 | 610 |
| 89.5 | 380 |
| 90.5 | 233 |
| 91.5 | 141 |
| 92.5 | 84 |
| 93.5 | 50 |
| 94.5 | 29 |
| 95.5 | 17 |
| 96.5 | 9 |
| 97.5 | 5 |
| 98.5 | 3 |
| 99.5 | 2 |
| 100.5 | 0 |
| 101.5 | 0 |
| 102.5 | 0 |
| 103.5 | 0 |
| 104.5 | 0 |
| 105.5 | 0 |
| 106.5 | 0 |
| 107.5 | 0 |
| 108.5 | 0 |
| 109.5 | 0 |
| 110.5 | 0 |
| 111.5 | 0 |
| 112.5 | 0 |
| 113.5 | 0 |
| 114.5 | 0 |
| 115.5 | 0 |
| 116.5 | 0 |
| 117.5 | 0 |
| 118.5 | 0 |
| 119.5 | 0 |
| 120.5 | 0 |
| 121.5 | 0 |
| 122.5 | 0 |
| 123.5 | 0 |
| 124.5 | 0 |
| 125.5 | 0 |
| 126.5 | 0 |
| 127.5 | 0 |
| 128.5 | 0 |
| 129.5 | 0 |
| 130.5 | 0 |
| 131.5 | 0 |
| 132.5 | 0 |
| 133.5 | 0 |
| 134.5 | 0 |
| 135.5 | 0 |
| 136.5 | 0 |
| 137.5 | 0 |
| 138.5 | 0 |
| 139.5 | 0 |
| 140.5 | 0 |
| 141.5 | 0 |
| 142.5 | 0 |
| 143.5 | 0 |
| 144.5 | 0 |
| 145.5 | 0 |
| 146.5 | 0 |
| 147.5 | 0 |
| 148.5 | 0 |
| 149.5 | 0 |
| 150.5 | 0 |
| 151.5 | 0 |
| 152.5 | 0 |
| 153.5 | 0 |
| 154.5 | 0 |
| 155.5 | 0 |
| 156.5 | 0 |
| 157.5 | 0 |
| 158.5 | 0 |
| 159.5 | 0 |
| 160.5 | 0 |
| 161.5 | 0 |
| 162.5 | 0 |
| 163.5 | 0 |
| 164.5 | 0 |
| 165.5 | 0 |
| 166.5 | 0 |
| 167.5 | 0 |
| 168.5 | 0 |
| 169.5 | 0 |
| 170.5 | 0 |
| 171.5 | 0 |
| 172.5 | 0 |
| 173.5 | 0 |
| 174.5 | 0 |
| 175.5 | 0 |
| 176.5 | 0 |
| 177.5 | 0 |
| 178.5 | 0 |
| 179.5 | 0 |
| 180.5 | 0 |
| 181.5 | 0 |
| 182.5 | 0 |
| 183.5 | 0 |
| 184.5 | 0 |
| 185.5 | 0 |
| 186.5 | 0 |
| 187.5 | 0 |
| 188.5 | 0 |
| 189.5 | 0 |
| 190.5 | 0 |
| 191.5 | 0 |
| 192.5 | 0 |
| 193.5 | 0 |
| 194.5 | 0 |
| 195.5 | 0 |
| 196.5 | 0 |
| 197.5 | 0 |
| 198.5 | 0 |
| 199.5 | 0 |
| 200.5 | 0 |
| 201.5 | 0 |
| 202.5 | 0 |
| 203.5 | 0 |
| 204.5 | 0 |
| 205.5 | 0 |
| 206.5 | 0 |
| 207.5 | 0 |
| 208.5 | 0 |
| 209.5 | 0 |
| 210.5 | 0 |
| 211.5 | 0 |
| 212.5 | 0 |
| 213.5 | 0 |
| 214.5 | 0 |
| 215.5 | 0 |
| 216.5 | 0 |
| 217.5 | 0 |
| 218.5 | 0 |
| 219.5 | 0 |
| 220.5 | 0 |
| 221.5 | 0 |
| 222.5 | 0 |
| 223.5 | 0 |
| 224.5 | 0 |
| 225.5 | 0 |
| 226.5 | 0 |
| 227.5 | 0 |
| 228.5 | 0 |
| 229.5 | 0 |
| 230.5 | 0 |
| 231.5 | 0 |
| 232.5 | 0 |
| 233.5 | 0 |
| 234.5 | 0 |
| 235.5 | 0 |
| 236.5 | 0 |
| 237.5 | 0 |
| 238.5 | 0 |
| 239.5 | 0 |
| 240.5 | 0 |
| 241.5 | 0 |
| 242.5 | 0 |
| 243.5 | 0 |
| 244.5 | 0 |
| 245.5 | 0 |
| 246.5 | 0 |
| 247.5 | 0 |
| 248.5 | 0 |
| 249.5 | 0 |
| 250.5 | 0 |
| 251.5 | 0 |
| 252.5 | 0 |
| 253.5 | 0 |
| 254.5 | 0 |
| 255.5 | 0 |
| 256.5 | 0 |
| 257.5 | 0 |
| 258.5 | 0 |
| 259.5 | 0 |
| 260.5 | 0 |
| 261.5 | 0 |
| 262.5 | 0 |
| 263.5 | 0 |
| 264.5 | 0 |
| 265.5 | 0 |
| 266.5 | 0 |
| 267.5 | 0 |
| 268.5 | 0 |
| 269.5 | 0 |
| 270.5 | 0 |
| 271.5 | 0 |
| 272.5 | 0 |
| 273.5 | 0 |
| 274.5 | 0 |
| 275.5 | 0 |
| 276.5 | 0 |
| 277.5 | 0 |
| 278.5 | 0 |
| 279.5 | 0 |
| 280.5 | 0 |
| 281.5 | 0 |
| 282.5 | 0 |
| 283.5 | 0 |
| 284.5 | 0 |
| 285.5 | 0 |
| 286.5 | 0 |
| 287.5 | 0 |
| 288.5 | 0 |
| 289.5 | 0 |
| 290.5 | 0 |
| 291.5 | 0 |
| 292.5 | 0 |
| 293.5 | 0 |
| 294.5 | 0 |
| 295.5 | 0 |
| 296.5 | 0 |
| 297.5 | 0 |
| 298.5 | 0 |
| 299.5 | 0 |
| 300.5 | 0 |
| 301.5 | 0 |
| 302.5 | 0 |
| 303.5 | 0 |
| 304.5 | 0 |
| 305.5 | 0 |
| 306.5 | 0 |
| 307.5 | 0 |
| 308.5 | 0 |
| 309.5 | 0 |
| 310.5 | 0 |
| 311.5 | 0 |
| 312.5 | 0 |
| 313.5 | 0 |
| 314.5 | 0 |
| 315.5 | 0 |
| 316.5 | 0 |
| 317.5 | 0 |
| 318.5 | 0 |
| 319.5 | 0 |
| 320.5 | 0 |
| 321.5 | 0 |
| 322.5 | 0 |
| 323.5 | 0 |
| 324.5 | 0 |
| 325.5 | 0 |
| 326.5 | 0 |
| 327.5 | 0 |
| 328.5 | 0 |
| 329.5 | 0 |
| 330.5 | 0 |
| 331.5 | 0 |
| 332.5 | 0 |
| 333.5 | 0 |
| 334.5 | 0 |
| 335.5 | 0 |
| 336.5 | 0 |
| 337.5 | 0 |
| 338.5 | 0 |
| 339.5 | 0 |
| 340.5 | 0 |
| 341.5 | 0 |
| 342.5 | 0 |
| 343.5 | 0 |
| 344.5 | 0 |
| 345.5 | 0 |
| 346.5 | 0 |
| 347.5 | 0 |
| 348.5 | 0 |
| 349.5 | 0 |
| 350.5 | 0 |
| 351.5 | 0 |
| 352.5 | 0 |
| 353.5 | 0 |
| 354.5 | 0 |
| 355.5 | 0 |
| 356.5 | 0 |
| 357.5 | 0 |
| 358.5 | 0 |
| 359.5 | 0 |
| 360.5 | 0 |
| 361.5 | 0 |
| 362.5 | 0 |
| 363.5 | 0 |
| 364.5 | 0 |
| 365.5 | 0 |
| 366.5 | 0 |
| 367.5 | 0 |
| 368.5 | 0 |
| 369.5 | 0 |
| 370.5 | 0 |
| 371.5 | 0 |
| 372.5 | 0 |
| 373.5 | 0 |
| 374.5 | 0 |
| 375.5 | 0 |
| 376.5 | 0 |
| 377.5 | 0 |
| 378.5 | 0 |
| 379.5 | 0 |
| 380.5 | 0 |
| 381.5 | 0 |
| 382.5 | 0 |
| 383.5 | 0 |
| 384.5 | 0 |
| 385.5 | 0 |
| 386.5 | 0 |
| 387.5 | 0 |
| 388.5 | 0 |
| 389.5 | 0 |
| 390.5 | 0 |
| 391.5 | 0 |
| 392.5 | 0 |
| 393.5 | 0 |
| 394.5 | 0 |
| 395.5 | 0 |
| 396.5 | 0 |
| 397.5 | 0 |
| 398.5 | 0 |
| 399.5 | 0 |
| 400.5 | 0 |
| 401.5 | 0 |
| 402.5 | 0 |
| 403.5 | 0 |
| 404.5 | 0 |
| 405.5 | 0 |
| 406.5 | 0 |
| 407.5 | 0 |
| 408.5 | 0 |
| 409.5 | 0 |
| 410.5 | 0 |
| 411.5 | 0 |
| 412.5 | 0 |
| 413.5 | 0 |
| 414.5 | 0 |
| 415.5 | 0 |
| 416.5 | 0 |
| 417.5 | 0 |
| 418.5 | 0 |
| 419.5 | 0 |
| 420.5 | 0 |
| 421.5 | 0 |
| 422.5 | 0 |
| 423.5 | 0 |
| 424.5 | 0 |
| 425.5 | 0 |
| 426.5 | 0 |
| 427.5 | 0 |
| 428.5 | 0 |
| 429.5 | 0 |
| 430.5 | 0 |
| 431.5 | 0 |
| 432.5 | 0 |
| 433.5 | 0 |
| 434.5 | 0 |
| 435.5 | 0 |
| 436.5 | 0 |
| 437.5 | 0 |
| 438.5 | 0 |
| 439.5 | 0 |
| 440.5 | 0 |
| 441.5 | 0 |
| 442.5 | 0 |
| 443.5 | 0 |
| 444.5 | 0 |
| 445.5 | 0 |
| 446.5 | 0 |
| 447.5 | 0 |
| 448.5 | 0 |
| 449.5 | 0 |
| 450.5 | 0 |
| 451.5 | 0 |
| 452.5 | 0 |
| 453.5 | 0 |
| 454.5 | 0 |
| 455.5 | 0 |
| 456.5 | 0 |
| 457.5 | 0 |
| 458.5 | 0 |
| 459.5 | 0 |
| 460.5 | 0 |
| 461.5 | 0 |
| 462.5 | 0 |
| 463.5 | 0 |
| 464.5 | 0 |
| 465.5 | 0 |
| 466.5 | 0 |
| 467.5 | 0 |
| 468.5 | 0 |
| 469.5 | 0 |
| 470.5 | 0 |
| 471.5 | 0 |
| 472.5 | 0 |
| 473.5 | 0 |
| 474.5 | 0 |
| 475.5 | 0 |
| 476.5 | 0 |
| 477.5 | 0 |
| 478.5 | 0 |
| 479.5 | 0 |
| 480.5 | 0 |
| 481.5 | 0 |
| 482.5 | 0 |
| 483.5 | 0 |
| 484.5 | 0 |
| 485.5 | 0 |
| 486.5 | 0 |
| 487.5 | 0 |
| 488.5 | 0 |
| 489.5 | 0 |
| 490.5 | 0 |
| 491.5 | 0 |
| 492.5 | 0 |
| 493.5 | 0 |
| 494.5 | 0 |
| 495.5 | 0 |
| 496.5 | 0 |
| 497.5 | 0 |
| 498.5 | 0 |
| 499.5 | 0 |
| 500.5 | 0 |
| 501.5 | 0 |
| 502.5 | 0 |
| 503.5 | 0 |
| 504.5 | 0 |
| 505.5 | 0 |
| 506.5 | 0 |
| 507.5 | 0 |
| 508.5 | 0 |
| 509.5 | 0 |
| 510.5 | 0 |
| 511.5 | 0 |
| 512.5 | 0 |
| 513.5 | 0 |
| 514.5 | 0 |
| 515.5 | 0 |
| 516.5 | 0 |
| 517.5 | 0 |
| 518.5 | 0 |
| 519.5 | 0 |
| 520.5 | 0 |
| 521.5 | 0 |
| 522.5 | 0 |
| 523.5 | 0 |
| 524.5 | 0 |
| 525.5 | 0 |
| 526.5 | 0 |
| 527.5 | 0 |
| 528.5 | 0 |
| 529.5 | 0 |
| 530.5 | 0 |
| 531.5 | 0 |
| 532.5 | 0 |
| 533.5 | 0 |
| 534.5 | 0 |
| 535.5 | 0 |
| 536.5 | 0 |
| 537.5 | 0 |
| 538.5 | 0 |
| 539.5 | 0 |
| 540.5 | 0 |
| 541.5 | 0 |
| 542.5 | 0 |
| 543.5 | 0 |
| 544.5 | 0 |
| 545.5 | 0 |
| 546.5 | 0 |
| 547.5 | 0 |
| 548.5 | 0 |
| 549.5 | 0 |
| 550.5 | 0 |
| 551.5 | 0 |
| 552.5 | 0 |
| 553.5 | 0 |
| 554.5 | 0 |
| 555.5 | 0 |
| 556.5 | 0 |
| 557.5 | 0 |
| 558.5 | 0 |
| 559.5 | 0 |
| 560.5 | 0 |
| 561.5 | 0 |
| 562.5 | 0 |
| 563.5 | 0 |
| 564.5 | 0 |
| 565.5 | 0 |
| 566.5 | 0 |
| 567.5 | 0 |
| 568.5 | 0 |
| 569.5 | 0 |
| 570.5 | 0 |
| 571.5 | 0 |
| 572.5 | 0 |
| 573.5 | 0 |
| 574.5 | 0 |
| 575.5 | 0 |
| 576.5 | 0 |
| 577.5 | 0 |
| 578.5 | 0 |
| 579.5 | 0 |
| 580.5 | 0 |
| 581.5 | 0 |
| 582.5 | 0 |
| 583.5 | 0 |
| 584.5 | 0 |
| 585.5 | 0 |
| 586.5 | 0 |
| 587.5 | 0 |
| 588.5 | 0 |
| 589.5 | 0 |
| 590.5 | 0 |
| 591.5 | 0 |
| 592.5 | 0 |
| 593.5 | 0 |
| 594.5 | 0 |
| 595.5 | 0 |
| 596.5 | 0 |
| 597.5 | 0 |
| 598.5 | 0 |
| 599.5 | 0 |
| 600.5 | 0 |
| 601.5 | 0 |
| 602.5 | 0 |
| 603.5 | 0 |
| 604.5 | 0 |
| 605.5 | 0 |
| 606.5 | 0 |
| 607.5 | 0 |
| 608.5 | 0 |
| 609.5 | 0 |
| 610.5 | 0 |
| 611.5 | 0 |
| 612.5 | 0 |
| 613.5 | 0 |
| 614.5 | 0 |
| 615.5 | 0 |
| 616.5 | 0 |
| 617.5 | 0 |
| 618.5 | 0 |
| 619.5 | 0 |
| 620.5 | 0 |
| 621.5 | 0 |
| 622.5 | 0 |
| 623.5 | 0 |
| 624.5 | 0 |
| 625.5 | 0 |
| 626.5 | 0 |
| 627.5 | 0 |
| 628.5 | 0 |
| 629.5 | 0 |
| 630.5 | 0 |
| 631.5 | 0 |
| 632.5 | 0 |
| 633.5 | 0 |
| 634.5 | 0 |
| 635.5 | 0 |
| 636.5 | 0 |
| 637.5 | 0 |
| 638.5 | 0 |
| 639.5 | 0 |
| 640.5 | 0 |
| 641.5 | 0 |
| 642.5 | 0 |
| 643.5 | 0 |
| 644.5 | 0 |
| 645.5 | 0 |
| 646.5 | 0 |
| 647.5 | 0 |
| 648.5 | 0 |
| 649.5 | 0 |
| 650.5 | 0 |
| 651.5 | 0 |
| 652.5 | 0 |
| 653.5 | 0 |
| 654.5 | 0 |
| 655.5 | 0 |
| 656.5 | 0 |
| 657.5 | 0 |
| 658.5 | 0 |
| 659.5 | 0 |
| 660.5 | 0 |
| 661.5 | 0 |
| 662.5 | 0 |
| 663.5 | 0 |
| 664.5 | 0 |
| 665.5 | 0 |
| 666.5 | 0 |
| 667.5 | 0 |
| 668.5 | 0 |
| 669.5 | 0 |
| 670.5 | 0 |
| 671.5 | 0 |
| 672.5 | 0 |
| 673.5 | 0 |
| 674.5 | 0 |
| 675.5 | 0 |
| 676.5 | 0 |
| 677.5 | 0 |
| 678.5 | 0 |
| 679.5 | 0 |
| 680.5 | 0 |
| 681.5 | 0 |
| 682.5 | 0 |
| 683.5 | 0 |
| 684.5 | 0 |
| 685.5 | 0 |
| 686.5 | 0 |
| 687.5 | 0 |
| 688.5 | 0 |
| 689.5 | 0 |
| 690.5 | 0 |
| 691.5 | 0 |
| 692.5 | 0 |
| 693.5 | 0 |
| 694.5 | 0 |
| 695.5 | 0 |
| 696.5 | 0 |
| 697.5 | 0 |
| 698.5 | 0 |
| 699.5 | 0 |
| 700.5 | 0 |
| 701.5 | 0 |
| 702.5 | 0 |
| 703.5 | 0 |
| 704.5 | 0 |
| 705.5 | 0 |
| 706.5 | 0 |
| 707.5 | 0 |
| 708.5 | 0 |
| 709.5 | 0 |
| 710.5 | 0 |
| 711.5 | 0 |
| 712.5 | 0 |
| 713.5 | 0 |
| 714.5 | 0 |
| 715.5 | 0 |
| 716.5 | 0 |
| 717.5 | 0 |
| 718.5 | 0 |
| 719.5 | 0 |
| 720.5 | 0 |
| 721.5 | 0 |
| 722.5 | 0 |
| 723.5 | 0 |
| 724.5 | 0 |
| 725.5 | 0 |
| 726.5 | 0 |
| 727.5 | 0 |
| 728.5 | 0 |
| 729.5 | 0 |
| 730.5 | 0 |
| 731.5 | 0 |
| 732.5 | 0 |
| 733.5 | 0 |
| 734.5 | 0 |
| 735.5 | 0 |
| 736.5 | 0 |
| 737.5 | 0 |
| 738.5 | 0 |
| 739.5 | 0 |
| 740.5 | 0 |
| 741.5 | 0 |
| 742.5 | 0 |
| 743.5 | 0 |
| 744.5 | 0 |
| 745.5 | 0 |
| 746.5 | 0 |
| 747.5 | 0 |
| 748.5 | 0 |
| 749.5 | 0 |
| 750.5 | 0 |
| 751.5 | 0 |
| 752.5 | 0 |
| 753.5 | 0 |
| 754.5 | 0 |
| 755.5 | 0 |
| 756.5 | 0 |
| 757.5 | 0 |
| 758.5 | 0 |
| 759.5 | 0 |
| 760.5 | 0 |
| 761.5 | 0 |
| 762.5 | 0 |
| 763.5 | 0 |
| 764.5 | 0 |
| 765.5 | 0 |
| 766.5 | 0 |
| 767.5 | 0 |
| 768.5 | 0 |
| 769.5 | 0 |
| 770.5 | 0 |
| 771.5 | 0 |
| 772.5 | 0 |
| 773.5 | 0 |
| 774.5 | 0 |
| 775.5 | 0 |
| 776.5 | 0 |
| 777.5 | 0 |
| 778.5 | 0 |
| 779.5 | 0 |
| 780.5 | 0 |
| 781.5 | 0 |
| 782.5 | 0 |
| 783.5 | 0 |
| 784.5 | 0 |
| 785.5 | 0 |
| 786.5 | 0 |
| 787.5 | 0 |
| 788.5 | 0 |
| 789.5 | 0 |
| 790.5 | 0 |
| 791.5 | 0 |
| 792.5 | 0 |
| 793.5 | 0 |
| 794.5 | 0 |
| 795.5 | 0 |
| 796.5 | 0 |
| 797.5 | 0 |
| 798.5 | 0 |
| 799.5 | 0 |
| 800.5 | 0 |
| 801.5 | 0 |
| 802.5 | 0 |
| 803.5 | 0 |
| 804.5 | 0 |
| 805.5 | 0 |
| 806.5 | 0 |
| 807.5 | 0 |
| 808.5 | 0 |
| 809.5 | 0 |
| 810.5 | 0 |
| 811.5 | 0 |
| 812.5 | 0 |
| 813.5 | 0 |
| 814.5 | 0 |
| 815.5 | 0 |
| 816.5 | 0 |
| 817.5 | 0 |
| 818.5 | 0 |
| 819.5 | 0 |
| 820.5 | 0 |
| 821.5 | 0 |
| 822.5 | 0 |
| 823.5 | 0 |
| 824.5 | 0 |
| 825.5 | 0 |
| 826.5 | 0 |
| 827.5 | 0 |
| 828.5 | 0 |
| 829.5 | 0 |
| 830.5 | 0 |
| 831.5 | 0 |
| 832.5 | 0 |
| 833.5 | 0 |
| 834.5 | 0 |
| 835.5 | 0 |
| 836.5 | 0 |
| 837.5 | 0 |
| 838.5 | 0 |
| 839.5 | 0 |
| 840.5 | 0 |
| 841.5 | 0 |
| 842.5 | 0 |
| 843.5 | 0 |
| 844.5 | 0 |
| 845.5 | 0 |
| 846.5 | 0 |
| 847.5 | 0 |
| 848.5 | 0 |
| 849.5 | 0 |
| 850.5 | 0 |
| 851.5 | 0 |
| 852.5 | 0 |
| 853.5 | 0 |
| 854.5 | 0 |
| 855.5 | 0 |
| 856.5 | 0 |
| 857.5 | 0 |
| 858.5 | 0 |
| 859.5 | 0 |
| 860.5 | 0 |
| 861.5 | 0 |
| 862.5 | 0 |
| 863.5 | 0 |
| 864.5 | 0 |
| 865.5 | 0 |
| 866.5 | 0 |
| 867.5 | 0 |
| 868.5 | 0 |
| 869.5 | 0 |
| 870.5 | 0 |
| 871.5 | 0 |
| 872.5 | 0 |
| 873.5 | 0 |
| 874.5 | 0 |
| 875.5 | 0 |
| 876.5 | 0 |
| 877.5 | 0 |
| 878.5 | 0 |
| 879.5 | 0 |
| 880.5 | 0 |
| 881.5 | 0 |
| 882.5 | 0 |
| 883.5 | 0 |
| 884.5 | 0 |
| 885.5 | 0 |
| 886.5 | 0 |
| 887.5 | 0 |
| 888.5 | 0 |
| 889.5 | 0 |
| 890.5 | 0 |
| 891.5 | 0 |
| 892.5 | 0 |
| 893.5 | 0 |
| 894.5 | 0 |
| 895.5 | 0 |
| 896.5 | 0 |
| 897.5 | 0 |
| 898.5 | 0 |
| 899.5 | 0 |
| 900.5 | 0 |
| 901.5 | 0 |
| 902.5 | 0 |
| 903.5 | 0 |
| 904.5 | 0 |
| 905.5 | 0 |
| 906.5 | 0 |
| 907.5 | 0 |
| 908.5 | 0 |
| 909.5 | 0 |
| 910.5 | 0 |
| 911.5 | 0 |
| 912.5 | 0 |
| 913.5 | 0 |
| 914.5 | 0 |
| 915.5 | 0 |
| 916.5 | 0 |
| 917.5 | 0 |
| 918.5 | 0 |
| 919.5 | 0 |
| 920.5 | 0 |
| 921.5 | 0 |
| 922.5 | 0 |
| 923.5 | 0 |
| 924.5 | 0 |
| 925.5 | 0 |
| 926.5 | 0 |
| 927.5 | 0 |
| 928.5 | 0 |
| 929.5 | 0 |
| 930.5 | 0 |
| 931.5 | 0 |
| 932.5 | 0 |
| 933.5 | 0 |
| 934.5 | 0 |
| 935.5 | 0 |
| 936.5 | 0 |
| 937.5 | 0 |
| 938.5 | 0 |
| 939.5 | 0 |
| 940.5 | 0 |
| 941.5 | 0 |
| 942.5 | 0 |
| 943.5 | 0 |
| 944.5 | 0 |
| 945.5 | 0 |
| 946.5 | 0 |
| 947.5 | 0 |
| 948.5 | 0 |
| 949.5 | 0 |
| 950.5 | 0 |
| 951.5 | 0 |
| 952.5 | 0 |
| 953.5 | 0 |
| 954.5 | 0 |
| 955.5 | 0 |
| 956.5 | 0 |
| 957.5 | 0 |
| 958.5 | 0 |
| 959.5 | 0 |
| 960.5 | 0 |
| 961.5 | 0 |
| 962.5 | 0 |
| 963.5 | 0 |
| 964.5 | 0 |
| 965.5 | 0 |
| 966.5 | 0 |
| 967.5 | 0 |
| 968.5 | 0 |
| 969.5 | 0 |
| 970.5 | 0 |
| 971.5 | 0 |
| 972.5 | 0 |
| 973.5 | 0 |
| 974.5 | 0 |
| 975.5 | 0 |
| 976.5 | 0 |
| 977.5 | 0 |
| 978.5 | 0 |
| 979.5 | 0 |
| 980.5 | 0 |
| 981.5 | 0 |
| 982.5 | 0 |
| 983.5 | 0 |
| 984.5 | 0 |
| 985.5 | 0 |
| 986.5 | 0 |
| 987.5 | 0 |
| 988.5 | 0 |
| 989.5 | 0 |
| 990.5 | 0 |
| 991.5 | 0 |
| 992.5 | 0 |
| 993.5 | 0 |
| 994.5 | 0 |
| 995.5 | 0 |
| 996.5 | 0 |
| 997.5 | 0 |
| 998.5 | 0 |
| 999.5 | 0 |
|  |  |
| Percentile | Size (nm) |
| 0 | 0 |
| 1 | 50.9 |
| 2 | 52.3 |
| 3 | 53.3 |
| 4 | 54 |
| 5 | 54.5 |
| 6 | 55.1 |
| 7 | 55.5 |
| 8 | 55.9 |
| 9 | 56.2 |
| 10 | 56.6 |
| 11 | 56.9 |
| 12 | 57.2 |
| 13 | 57.4 |
| 14 | 57.7 |
| 15 | 58 |
| 16 | 58.2 |
| 17 | 58.4 |
| 18 | 58.7 |
| 19 | 58.9 |
| 20 | 59.1 |
| 21 | 59.3 |
| 22 | 59.5 |
| 23 | 59.7 |
| 24 | 59.9 |
| 25 | 60.1 |
| 26 | 60.2 |
| 27 | 60.4 |
| 28 | 60.6 |
| 29 | 60.8 |
| 30 | 60.9 |
| 31 | 61.1 |
| 32 | 61.3 |
| 33 | 61.4 |
| 34 | 61.6 |
| 35 | 61.8 |
| 36 | 61.9 |
| 37 | 62.1 |
| 38 | 62.2 |
| 39 | 62.4 |
| 40 | 62.6 |
| 41 | 62.7 |
| 42 | 62.9 |
| 43 | 63 |
| 44 | 63.2 |
| 45 | 63.3 |
| 46 | 63.5 |
| 47 | 63.6 |
| 48 | 63.8 |
| 49 | 63.9 |
| 50 | 64.1 |
| 51 | 64.2 |
| 52 | 64.4 |
| 53 | 64.6 |
| 54 | 64.7 |
| 55 | 64.9 |
| 56 | 65 |
| 57 | 65.2 |
| 58 | 65.3 |
| 59 | 65.5 |
| 60 | 65.7 |
| 61 | 65.8 |
| 62 | 66 |
| 63 | 66.1 |
| 64 | 66.3 |
| 65 | 66.5 |
| 66 | 66.7 |
| 67 | 66.8 |
| 68 | 67 |
| 69 | 67.2 |
| 70 | 67.4 |
| 71 | 67.5 |
| 72 | 67.7 |
| 73 | 67.9 |
| 74 | 68.1 |
| 75 | 68.3 |
| 76 | 68.5 |
| 77 | 68.7 |
| 78 | 68.9 |
| 79 | 69.1 |
| 80 | 69.4 |
| 81 | 69.6 |
| 82 | 69.9 |
| 83 | 70.1 |
| 84 | 70.4 |
| 85 | 70.6 |
| 86 | 70.9 |
| 87 | 71.2 |
| 88 | 71.6 |
| 89 | 71.9 |
| 90 | 72.3 |
| 91 | 72.6 |
| 92 | 73.1 |
| 93 | 73.6 |
| 94 | 74.1 |
| 95 | 74.7 |
| 96 | 75.4 |
| 97 | 76.3 |
| 98 | 77.5 |
| 99 | 79.4 |
| 100 | 662 |
