# Supplementary material for: Exosomal miR-218-5p/miR-363-3p from Endothelial Progenitor Cells Ameliorate Myocardial Infarction by Targeting the p53/JMY Signaling Pathway
Source: Oxid Med Cell Longev. 2021 Jul 16;2021:5529430. doi: 10.1155/2021/5529430 (PMC8302385; doi:10.1155/2021/5529430)
Supplement: Supplementary Materials — Supplemental Figure 1: characterization of EPC-Exos. (A) Transmission electron microscopy analysis of EPC-Exos in sparse regions. Scale bar: 100 nm. (B) The particle diameter size distribution of EPC-Exos. (C) Western analysis of the surface proteins of exosomes (CD63, Alix, TSG101, and Calnexin). (D) PKH67 staining of EPC-Exos. Supplemental Figure 2: reads statistics for miRNA sequencing data in EPCs and EPC-Exos. (A) Length distribution of small RNAs in EPCs and EPC-Exos. (B) New miRNAs were discovered using miRDeep2. Supplemental Figure 3: Gene Ontology (GO) enrichment and KEGG pathway analysis of differentially expressed miRNAs in EPC-Exos vs EPCs. (A) KEGG pathway analysis of the upregulated differentially expressed miRNAs (left panel) and downregulated miRNAs (right panel) in EPC-Exos vs. EPCs. The P value (ease-score, Fisher's P value, or hypergeometric P value, cutoff at 0.05) denotes the significance of the pathway correlated with the conditions. The lower the P value is, the more significant the pathway is. (B) Gene Ontology (GO) enrichment analysis of upregulated differentially expressed miRNAs in EPC-Exos vs. EPCs. The top ten enrichment score counts in the GO biological process classification for biological process, cellular components, and molecular function are listed. (C) Gene Ontology (GO) enrichment analysis of downregulated differentially expressed miRNAs in EPC-Exos vs. EPCs. Supplemental Figure 4: characterization of EPC-Exos treated with GW4869. (A) Transmission electron microscopy analysis of EPC-Exos and EPC-Exos treated with GW4869 in sparse regions. Scale bar: 100 nm. (B) The particle diameter size distribution and concentration of EPC-Exos with and without GW4869 treatment. (C, D) The relative expression of miR-218-5p and miR-363-3p in EPCs and EPC-Exos with and without GW4869 treatment. ∗∗P < 0.01, GW4869 versus control. Supplemental Figure 5: function analysis of EPC-Exos to CF proliferation and angiogenesis treated with or without GW486 [file 5529430.f1.zip › Suppmental Tables_Accepted.docx]

**Supplemental Tables**

**Supplemental Table 1**. List of primers for qRT-PCR to detect the 16 differentially expressed miRNAs.

| ID | Sequence (5’-3’) | Tm (℃) |
| --- | --- | --- |
| U6 F | CTCGCTTCGGCAGCACA | 60.42 |
| U6 R | AACGCTTCACGAATTTGCGT | 59.69 |
| All R | CTCAACTGGTGTCGTGGA | 51.5 |
| hsa-miR-218-5p | TTGTGCTTGATCTAACCATGT |  |
| hsa-miR-218-5p RT | CTCAACTGGTGTCGTGGAGTCGGCAATTCAGTTGAGACATGGTT | 86.3 |
| hsa-miR-218-5p F | ACACTCCAGCTGGGTTGTGCTTGATCTAACCA | 75.9 |
| hsa-miR-1-3p | TGGAATGTAAAGAAGTATGTAT |  |
| hsa-miR-1-3p RT | CTCAACTGGTGTCGTGGAGTCGGCAATTCAGTTGAGATACATAC | 82.7 |
| hsa-miR-1-3p F | ACACTCCAGCTGGGTGGAATGTAAAGAAGTAT | 70.8 |
| hsa-miR-1246 | AATGGATTTTTGGAGCAGG |  |
| hsa-miR-1246 RT | CTCAACTGGTGTCGTGGAGTCGGCAATTCAGTTGAGCCTGCT | 87.3 |
| hsa-miR-1246 F | ACACTCCAGCTGGGAATGGATTTTTGGAGCA | 77.2 |
| hsa-miR-122-5p | TGGAGTGTGACAATGGTGTTTG |  |
| hsa-miR-122-5p RT | CTCAACTGGTGTCGTGGAGTCGGCAATTCAGTTGAGCAAACACC | 87.5 |
| hsa-miR-122-5p F | ACACTCCAGCTGGGTGGAGTGTGACAATGGTG | 78.6 |
| hsa-miR-451a | AAACCGTTACCATTACTGAGTT |  |
| hsa-miR-451a RT | CTCAACTGGTGTCGTGGAGTCGGCAATTCAGTTGAGAACTCAGT | 85.0 |
| hsa-miR-451a F | ACACTCCAGCTGGGAAACCGTTACCATTACTG | 74.6 |
| hsa-miR-6087 | TGAGGCGGGGGGGCGAGC |  |
| hsa-miR-6087 RT | CTCAACTGGTGTCGTGGAGTCGGCAATTCAGTTGAGGCTCGC | 88.6 |
| hsa-miR-6087 F | ACACTCCAGCTGGGTGAGGCGGGGGGGCGA | 87.7 |
| hsa-miR-363-3p | AATTGCACGGTATCCATCTGTA |  |
| hsa-miR-363-3p RT | CTCAACTGGTGTCGTGGAGTCGGCAATTCAGTTGAGTACAGATG | 84.6 |
| hsa-miR-363-3p F | ACACTCCAGCTGGGAATTGCACGGTATCCATC | 78.1 |
| hsa-miR-486-5p | TCCTGTACTGAGCTGCCCCGAG |  |
| hsa-miR-486-5p RT | CTCAACTGGTGTCGTGGAGTCGGCAATTCAGTTGAGCTCGGGGC | 90.8 |
| hsa-miR-486-5p F | ACACTCCAGCTGGGTCCTGTACTGAGCTGCCC | 79.5 |
| hsa-miR-500a-3p | ATGCACCTGGGCAAGGATTCTG |  |
| hsa-miR-500a-3p RT | CTCAACTGGTGTCGTGGAGTCGGCAATTCAGTTGAGCAGAATCC | 87.1 |
| hsa-miR-500a-3p F | ACACTCCAGCTGGGATGCACCTGGGCAAGGAT | 82.2 |
| hsa-miR-29b-3p | TAGCACCATTTGAAATCAGTGTT |  |
| hsa-miR-29b-3p RT | CTCAACTGGTGTCGTGGAGTCGGCAATTCAGTTGAGAACACTGA | 86.1 |
| hsa-miR-29b-3p F | ACACTCCAGCTGGGTAGCACCATTTGAAATCA | 75.7 |
| hsa-miR-362-5p | AATCCTTGGAACCTAGGTGTGAGT |  |
| hsa-miR-362-5p RT | CTCAACTGGTGTCGTGGAGTCGGCAATTCAGTTGAGACTCACACC | 87.3 |
| hsa-miR-362-5p F | ACACTCCAGCTGGGAATCCTTGGAACCTAGGTG | 77.6 |
| hsa-miR-221-5p | ACCTGGCATACAATGTAGATTT |  |
| hsa-miR-221-5p RT | CTCAACTGGTGTCGTGGAGTCGGCAATTCAGTTGAGAAATCTAC | 83.3 |
| hsa-miR-221-5p F | ACACTCCAGCTGGGACCTGGCATACAATGTAG | 75.4 |
| hsa-miR-21-3p | CAACACCAGTCGATGGGCTGT |  |
| hsa-miR-21-3p RT | CTCAACTGGTGTCGTGGAGTCGGCAATTCAGTTGAGACAGCCCA | 89.0 |
| hsa-miR-21-3p F | ACACTCCAGCTGGGCAACACCAGTCGATGGGC | 83.4 |
| hsa-miR-374a-3p | CTTATCAGATTGTATTGTAATT |  |
| hsa-miR-374a-3p RT | CTCAACTGGTGTCGTGGAGTCGGCAATTCAGTTGAGAATTACAA | 83.9 |
| hsa-miR-374a-3p F | ACACTCCAGCTGGGCTTATCAGATTGTATTGT | 71.5 |
| hsa-miR-365a-5p | AGGGACTTTTGGGGGCAGATGTG |  |
| hsa-miR-365a-5p RT | CTCAACTGGTGTCGTGGAGTCGGCAATTCAGTTGAGCACATCTG | 87.3 |
| hsa-miR-365a-5p F | ACACTCCAGCTGGGAGGGACTTTTGGGGGCAGA | 83.3 |
| hsa-miR-181a-3p | ACCATCGACCGTTGATTGTACC |  |
| hsa-miR-181a-3p RT | CTCAACTGGTGTCGTGGAGTCGGCAATTCAGTTGAGGGTACAAT | 85.5 |
| hsa-miR-181a-3p F | ACACTCCAGCTGGGACCATCGACCGTTGATTG | 80.4 |
| GAPDH F | TGTTCGTCATGGGTGTGAAC | 56.7 |
| GAPDH R | ATGGCATGGACTGTGGTCAT | 57.8 |
| ACTA2 F | CCCTTGAGAAGAGTTACGAGTT | 55.1 |
| ACTA2 R | ATGATGCTGTTGTAGGTGGTT | 54.5 |
| Vimentin F | CCTTGAACGCAAAGTGGAAT | 57.8 |
| Vimentin R | AGGTCAGGCTTGGAAACATC | 56.4 |
| CD31 F | CCTGCGGTATTCAAAGACAAC | 57.5 |
| CD31 R | GGGACCAGATCCTTCATTCAC | 58.1 |
| KDR F | AGAGTGGCAGTGAGCAAAGG | 57.6 |
| KDR R | CATAGACATAAATGACCGAGGC | 56.9 |
| TP53 F | CAGCACATGACGGAGGTTGT | 58.8 |
| TP53 R | TCATCCAAATACTCCACACGC | 58.3 |
| JMY F | AGCAGGAAATCGACACTCTGT | 56.3 |
| JMY R | TCCTCGGGATCATCGGTCTC | 61.8 |

**Supplemental Table 2**. List of primers for construction of the wild-type and mutant p53 promoter and JMY 3’UTR used in the luciferase assays.

| **ID** | **Sequence (5’-3’)** |
| --- | --- |
| JMY-P1 | JMY-F1:CCGCTCGAGGCAGGTTATCACTTTCCAGTCGTTC |
|  | JMY-R1:ATTTGCGGCCGCTAAAAGCACGGAAACTTCAAAACCT |
|  | JMY-MUT-F1:ACATGAATTTTACATCGTGCCAGTTCCTGACTGCAAT |
|  | JMY-MUT-R1:ATTGCAGTCAGGAACTGGCACGATGTAAAATTCATGT |
| JMY-P2 | JMY-F2:CCGCTCGAGTAAATGATGCTACCCATACAGTGAC |
|  | JMY-R2:ATTTGCGGCCGCAAAAGGCAACAGTCAAAACGA |
|  | JMY-MUT-F2:CCATTTACTAGCTTTCGTACCGATTATAAAAGGTAG |
|  | JMY-MUT-R2:CTACCTTTTATAATCGGTACGAAAGCTAGTAAATGG |
| TP53 | TP53-F: GGGGTACCACCGAGTCCCGCGGTAATTC |
|  | TP53-R: CCCTCGAGATCTCCTTCACAACCCTTATCACTC |
|  | TP53-MUT-F: CCGCGGTAATTCTTATCAGCTCTGCACCGCCCC |
|  | TP53-MUT-R: GGGGCGGTGCAGAGCTGATAAGAATTACCGCGG |

**Supplemental Table 3**. List of miRNA mimic and inhibitor.

| **miRNA** | **Sequence** |
| --- | --- |
| **miR-218-5p mimic** | 5’-UUGUGCUUGAUCUAACCAUGU-3’ |
|  | 3’-AUGGUUAGAUCAAGCACAAUU-5’ |
| **miR-218-5p inhibitor** | 5’-ACAUGGUUAGAUCAAGCACAA-3’ |
| **miR-363-3p mimic** | 5’-AAUUGCACGGUAUCCAUCUGUA-3’ |
|  | 3’-CAGAUGGAUACCGUGCAAUUUU-5’ |
| **miR-363-3p inhibitor** | 5’-UACAGAUGGAUACCGUGCAAUU-3’ |
| **cel-miR-39 mimic** | 5'-UCACCGGGUGUAAAUCAGCUUG-3' |
|  | 3'-AGCUGAUUUACACCCGGUGAUU-5' |
| **miR-6087 mimic** | 5’-UGAGGCGGGGGGGCGAGC-3’ |
|  | 3’-UCGCCCCCCCGCCUCAUU-5’ |
| **miR-6087 inhibitor** | 5’-GCUCGCCCCCCCGCCUCA-3’ |

**Supplemental Table 4.** Effect of EPC-Exos containing miR-218-5p mimic or miR-363-3p mimic on the cardiac ultrasonography index of LCA ligation-induced myocardial infarction rats.

| **Groups** | **Sham** | **Model** | **mimic-218-Exo** | **mimic-363-Exo** |
| --- | --- | --- | --- | --- |
| HR (bpm) | 333±11.6 | 265±13.9 | 314±13.9 (p=0.555) | 315±16.5 (p=0.532) |
| LVESd (mm) | 3.741±0.103 | 5.226±0.221 | 4.593±0.276 (p=0.598) | 4.325±0.215 (p=0.515) |
| LVEDd (mm) | 5.954±0.297 | 7.023±0.265 | 6.483±0.293 (p=0.810) | 6.443±0.331 (p=0.767) |
| LVIDs (µL) | 94.103±2.084 | 108.871±1.740 | 102.014±1.972 (p=0.878) | 94.874±2.256 (p=0.656) |
| LVIDd (µL) | 246.593±12.001 | 217.715±7.273 | 224.714±10.788 (p=0.899) | 231.734±7.194 (p=0.766) |
| SV (µL) | 152.49±2.990 | 108.844±1.787 | 122.7±4.865 (p=0.599) | 136.86±2.487 (p=0.419) |
| LVEF (%) | 73.812±1.852 | 52.994±1.829 | 60.488±2.648 (p=0.633) | 65.192±2.522 (p=0.472) |
| FS (%) | 37.168±2.620 | 25.587±2.528 | 29.153±2.420 (p=0.719) | 32.872±1.444 (p=0.473) |
| CO (mL/min) | 50.77917±2.486 | 28.84366±1.986 | 38.5278±1.272 (p=0.305) | 43.1109±2.048 (p=0.515) |
| LVPWT (mm) | 2.215±0.112 | 2.208±0.138 | 2.227±0.109 (p=0.882) | 2.209±0.131 (p=0.793) |
| LVAWT (mm) | 2.224±0.153 | 1.762±0.124 | 1.969±0.147 (p=0.469) | 2.073±0.163 (p=0.526) |

**Note:** HR: heart rate; bpm: beat per minute; LVESd: left ventricular end systolic diameter; LVEDd: left ventricular end diastolic diameter; LVIDs: left ventricular internal diameter end systole; LVIDd: left ventricular internal diameter end diastole; SV: stroke volume; LVEF: left ventricular ejection fraction; FS: fractional shortening; CO: cardiac output; LVPWT: left ventricular posterior wall thickness; LVAWT: left ventricular anterior wall thickness. The P value refers to mimic-218-Exo or mimic-363-Exo vs. the model group.

**Supplemental Table 5.** Reads statistics for cell and exosome samples.

| Sample Name | Clean Reads | Adapter-trimmed Reads (length >= 15nt) | Reads aligned to known Human pre-miRNA in miRBase21 |
| --- | --- | --- | --- |
| Cell | 7,342,859 | 6,997,238 | 4,691,984 |
| Exosomes | 8,898,449 | 8,813,698 | 127,888 |

**Supplemental Table 6.** Novel miRNAs predicted by miRDeep2.

| **Provisional id** | **Precursor coordinate** | **miRDeep2 score** | **True positive** | **p-value** | **Consensus mature sequence** |
| --- | --- | --- | --- | --- | --- |
| [**chr16_6276**](file:///G:\论文撰写\Processing\稿件修改\PaperData%20测序\Novel_miRNAs\novel_miRNA_pdf\chr16_6276.pdf) | chr16:50869453..50869519:- | 11939.6 | 64 +/- 14% | no | ggaauaccgggugcuguaggcuu |
| [**chr9_24807**](file:///G:\论文撰写\Processing\稿件修改\PaperData%20测序\Novel_miRNAs\novel_miRNA_pdf\chr9_24807.pdf) | chr9:4850308..4850366:+ | 6108.9 | 64 +/- 14% | yes | guacaguacugugauaacugaa |
| [**chr17_7389**](file:///G:\论文撰写\Processing\稿件修改\PaperData%20测序\Novel_miRNAs\novel_miRNA_pdf\chr17_7389.pdf) | chr17:48642604..48642666:- | 147.2 | 64 +/- 14% | no | gcgacugccucccugcugugagc |
| [**chr6_21703**](file:///G:\论文撰写\Processing\稿件修改\PaperData%20测序\Novel_miRNAs\novel_miRNA_pdf\chr6_21703.pdf) | chr6:28863681..28863719:- | 120.6 | 64 +/- 14% | no | uccccggcaccuccagcu |
| [**chr8_24540**](file:///G:\论文撰写\Processing\稿件修改\PaperData%20测序\Novel_miRNAs\novel_miRNA_pdf\chr8_24540.pdf) | chr8:98393666..98393724:- | 66.9 | 64 +/- 14% | yes | uuauccuccaguagacuaggga |
| [**chr2_14626**](file:///G:\论文撰写\Processing\稿件修改\PaperData%20测序\Novel_miRNAs\novel_miRNA_pdf\chr2_14626.pdf) | chr2:231550178..231550240:+ | 63 | 64 +/- 14% | yes | gaguucugggcuguagua |
| [**chr19_8501**](file:///G:\论文撰写\Processing\稿件修改\PaperData%20测序\Novel_miRNAs\novel_miRNA_pdf\chr19_8501.pdf) | chr19:53762358..53762419:+ | 57.9 | 64 +/- 14% | yes | aaagugcauccuuuuagagugu |
| [**chr14_4360**](file:///G:\论文撰写\Processing\稿件修改\PaperData%20测序\Novel_miRNAs\novel_miRNA_pdf\chr14_4360.pdf) | chr14:94783821..94783891:+ | 46.9 | 64 +/- 14% | no | cuuggaccugaaggugga |
| [**chr6_21343**](file:///G:\论文撰写\Processing\稿件修改\PaperData%20测序\Novel_miRNAs\novel_miRNA_pdf\chr6_21343.pdf) | chr6:142013664..142013723:+ | 37 | 64 +/- 14% | yes | uauauacaagcacacacacaaau |
| [**chr1_11330**](file:///G:\论文撰写\Processing\稿件修改\PaperData%20测序\Novel_miRNAs\novel_miRNA_pdf\chr1_11330.pdf) | chr1:218541837..218541899:- | 28 | 64 +/- 14% | yes | gaguuucucugaaccuguagagc |
| [**chr18_7872**](file:///G:\论文撰写\Processing\稿件修改\PaperData%20测序\Novel_miRNAs\novel_miRNA_pdf\chr18_7872.pdf) | chr18:74148958..74149017:+ | 23.7 | 64 +/- 14% | no | aacgcugcgaccuagauguauucu |
| [**chr17_7136**](file:///G:\论文撰写\Processing\稿件修改\PaperData%20测序\Novel_miRNAs\novel_miRNA_pdf\chr17_7136.pdf) | chr17:17237109..17237167:- | 17.6 | 64 +/- 14% | yes | cugguucugccagcuccccuga |
| [**chr9_25045**](file:///G:\论文撰写\Processing\稿件修改\PaperData%20测序\Novel_miRNAs\novel_miRNA_pdf\chr9_25045.pdf) | chr9:83714218..83714274:+ | 12.4 | 64 +/- 14% | yes | acaaaacugcaguuacuuuugu |
| [**chr2_15598**](file:///G:\论文撰写\Processing\稿件修改\PaperData%20测序\Novel_miRNAs\novel_miRNA_pdf\chr2_15598.pdf) | chr2:219294125..219294185:- | 11.2 | 64 +/- 14% | yes | uugcauagucacaaaagugauc |
| [**chr3_16536**](file:///G:\论文撰写\Processing\稿件修改\PaperData%20测序\Novel_miRNAs\novel_miRNA_pdf\chr3_16536.pdf) | chr3:186787299..186787359:+ | 9.3 | 68 +/- 13% | yes | aaagcaggauucagacuacaauau |
| [**chr3_16591**](file:///G:\论文撰写\Processing\稿件修改\PaperData%20测序\Novel_miRNAs\novel_miRNA_pdf\chr3_16591.pdf) | chr3:197293885..197293944:+ | 9.2 | 68 +/- 13% | yes | aguaaguggcacucugucuucu |
| [**chr6_21492**](file:///G:\论文撰写\Processing\稿件修改\PaperData%20测序\Novel_miRNAs\novel_miRNA_pdf\chr6_21492.pdf) | chr6:10722842..10722903:- | 8.2 | 69 +/- 12% | yes | ccggaguuggaccugggacuug |
| [**chr5_19827**](file:///G:\论文撰写\Processing\稿件修改\PaperData%20测序\Novel_miRNAs\novel_miRNA_pdf\chr5_19827.pdf) | chr5:31515423..31515485:- | 7.8 | 71 +/- 11% | yes | ucuugggccccaccccuggaga |
| [**chr1_9081**](file:///G:\论文撰写\Processing\稿件修改\PaperData%20测序\Novel_miRNAs\novel_miRNA_pdf\chr1_9081.pdf) | chr1:19910424..19910514:+ | 7 | 71 +/- 11% | yes | uaauguaguugccacuaggaga |
| [**chr12_2824**](file:///G:\论文撰写\Processing\稿件修改\PaperData%20测序\Novel_miRNAs\novel_miRNA_pdf\chr12_2824.pdf) | chr12:26804109..26804173:- | 6.9 | 73 +/- 10% | yes | ucccuuuucugguaguucuca |
| [**chr16_5990**](file:///G:\论文撰写\Processing\稿件修改\PaperData%20测序\Novel_miRNAs\novel_miRNA_pdf\chr16_5990.pdf) | chr16:81533905..81533962:+ | 6.6 | 73 +/- 10% | yes | cuuccaucuccaucaccuugagc |
| [**chr14_4040**](file:///G:\论文撰写\Processing\稿件修改\PaperData%20测序\Novel_miRNAs\novel_miRNA_pdf\chr14_4040.pdf) | chr14:19097814..19097855:+ | 5.8 | 77 +/- 8% | yes | cgcacuugcggccccggc |
| [**chr2_15195**](file:///G:\论文撰写\Processing\稿件修改\PaperData%20测序\Novel_miRNAs\novel_miRNA_pdf\chr2_15195.pdf) | chr2:131659110..131659198:- | 5.4 | 77 +/- 8% | yes | agcggggccucacgauc |
| [**chr13_3371**](file:///G:\论文撰写\Processing\稿件修改\PaperData%20测序\Novel_miRNAs\novel_miRNA_pdf\chr13_3371.pdf) | chr13:26685335..26685399:+ | 5.4 | 77 +/- 8% | yes | uaugugccuaguggcugcuguc |
| [**chr3_16836**](file:///G:\论文撰写\Processing\稿件修改\PaperData%20测序\Novel_miRNAs\novel_miRNA_pdf\chr3_16836.pdf) | chr3:51928894..51928956:- | 5.3 | 77 +/- 8% | yes | aggaaacucugguagagga |
| [**chr21_12886**](file:///G:\论文撰写\Processing\稿件修改\PaperData%20测序\Novel_miRNAs\novel_miRNA_pdf\chr21_12886.pdf) | chr21:9366341..9366392:+ | 5.3 | 77 +/- 8% | yes | ucucugggccugugucuu |
| [**chr20_12170**](file:///G:\论文撰写\Processing\稿件修改\PaperData%20测序\Novel_miRNAs\novel_miRNA_pdf\chr20_12170.pdf) | chr20:50242553..50242600:- | 5.3 | 77 +/- 8% | yes | aggaggaagguggggaca |
| [**chr10_399**](file:///G:\论文撰写\Processing\稿件修改\PaperData%20测序\Novel_miRNAs\novel_miRNA_pdf\chr10_399.pdf) | chr10:112855014..112855088:+ | 5.2 | 77 +/- 8% | yes | aaggagcuggagaagcu |
| [**chr3_16834**](file:///G:\论文撰写\Processing\稿件修改\PaperData%20测序\Novel_miRNAs\novel_miRNA_pdf\chr3_16834.pdf) | chr3:51878044..51878103:- | 5.2 | 77 +/- 8% | yes | aggaaacucugguagagga |
| [**chr3_16242**](file:///G:\论文撰写\Processing\稿件修改\PaperData%20测序\Novel_miRNAs\novel_miRNA_pdf\chr3_16242.pdf) | chr3:124325437..124325500:+ | 4.8 | 51 +/- 11% | yes | aggaggaagguggggaca |
| [**chr18_7952**](file:///G:\论文撰写\Processing\稿件修改\PaperData%20测序\Novel_miRNAs\novel_miRNA_pdf\chr18_7952.pdf) | chr18:21829014..21829075:- | 4.8 | 51 +/- 11% | yes | uggaauguaaagaaguauguau |
| [**chr17_6685**](file:///G:\论文撰写\Processing\稿件修改\PaperData%20测序\Novel_miRNAs\novel_miRNA_pdf\chr17_6685.pdf) | chr17:27961771..27961818:+ | 4.7 | 51 +/- 11% | yes | uccgggcaacaaagugaga |
| [**chr5_19609**](file:///G:\论文撰写\Processing\稿件修改\PaperData%20测序\Novel_miRNAs\novel_miRNA_pdf\chr5_19609.pdf) | chr5:165781852..165781889:+ | 4.6 | 51 +/- 11% | yes | aaguguuguguucagaggc |
| [**chr2_15240**](file:///G:\论文撰写\Processing\稿件修改\PaperData%20测序\Novel_miRNAs\novel_miRNA_pdf\chr2_15240.pdf) | chr2:135335273..135335328:- | 4.6 | 51 +/- 11% | yes | aauagugucuagaauaucuuga |
| [**chr20_11907**](file:///G:\论文撰写\Processing\稿件修改\PaperData%20测序\Novel_miRNAs\novel_miRNA_pdf\chr20_11907.pdf) | chr20:407823..407880:- | 4.1 | 51 +/- 11% | no | acagcgucgagccgcuggggacgg |
| [**chr19_8338**](file:///G:\论文撰写\Processing\稿件修改\PaperData%20测序\Novel_miRNAs\novel_miRNA_pdf\chr19_8338.pdf) | chr19:35161526..35161585:+ | 3.6 | 3 +/- 6% | yes | ucaugucugaaccaaugagagc |
| [**chr19_8267**](file:///G:\论文撰写\Processing\稿件修改\PaperData%20测序\Novel_miRNAs\novel_miRNA_pdf\chr19_8267.pdf) | chr19:15289643..15289711:+ | 3.3 | 3 +/- 6% | yes | ccaaaacagcaguuacuuuugc |
| [**chr5_20190**](file:///G:\论文撰写\Processing\稿件修改\PaperData%20测序\Novel_miRNAs\novel_miRNA_pdf\chr5_20190.pdf) | chr5:102787498..102787557:- | 3.2 | 3 +/- 6% | yes | acagauuuccugggcuuagugaaa |
| [**chr5_19255**](file:///G:\论文撰写\Processing\稿件修改\PaperData%20测序\Novel_miRNAs\novel_miRNA_pdf\chr5_19255.pdf) | chr5:102787502..102787561:+ | 3.1 | 3 +/- 6% | yes | acagauuuccugggcuuagugaaa |
| [**chr18_7848**](file:///G:\论文撰写\Processing\稿件修改\PaperData%20测序\Novel_miRNAs\novel_miRNA_pdf\chr18_7848.pdf) | chr18:66630337..66630397:+ | 2.7 | 2 +/- 5% | yes | ugagugugcgugugugagugugc |
| [**chr8_24333**](file:///G:\论文撰写\Processing\稿件修改\PaperData%20测序\Novel_miRNAs\novel_miRNA_pdf\chr8_24333.pdf) | chr8:41201559..41201634:- | 2.5 | 2 +/- 5% | yes | ucaggcucaguccccucc |
| [**chr16_5794**](file:///G:\论文撰写\Processing\稿件修改\PaperData%20测序\Novel_miRNAs\novel_miRNA_pdf\chr16_5794.pdf) | chr16:34163598..34163652:+ | 2.4 | 2 +/- 5% | yes | ucccggucgccgcgguucgccg |
| [**chr1_9947**](file:///G:\论文撰写\Processing\稿件修改\PaperData%20测序\Novel_miRNAs\novel_miRNA_pdf\chr1_9947.pdf) | chr1:201888877..201888955:+ | 2.3 | 2 +/- 5% | yes | ccggcuugguccugcggucccu |
| [**chr9_25450**](file:///G:\论文撰写\Processing\稿件修改\PaperData%20测序\Novel_miRNAs\novel_miRNA_pdf\chr9_25450.pdf) | chr9:35096058..35096110:- | 2.3 | 2 +/- 5% | yes | cugccuguacaugugaacguu |
| [**chr13_3738**](file:///G:\论文撰写\Processing\稿件修改\PaperData%20测序\Novel_miRNAs\novel_miRNA_pdf\chr13_3738.pdf) | chr13:38683990..38684074:- | 2.2 | 2 +/- 5% | yes | acuggaggucagacugg |
| [**chr9_25423**](file:///G:\论文撰写\Processing\稿件修改\PaperData%20测序\Novel_miRNAs\novel_miRNA_pdf\chr9_25423.pdf) | chr9:32456302..32456370:- | 2.2 | 2 +/- 5% | yes | aaaagcuguccacuguagaguu |
| [**chr5_20214**](file:///G:\论文撰写\Processing\稿件修改\PaperData%20测序\Novel_miRNAs\novel_miRNA_pdf\chr5_20214.pdf) | chr5:108748635..108748690:- | 2.2 | 2 +/- 5% | yes | cgccgcuuucugggcucgcuc |
| [**chr19_8528**](file:///G:\论文撰写\Processing\稿件修改\PaperData%20测序\Novel_miRNAs\novel_miRNA_pdf\chr19_8528.pdf) | chr19:57439111..57439174:+ | 2.2 | 2 +/- 5% | yes | uuaguggcucccucugccugca |
| [**chr10_126**](file:///G:\论文撰写\Processing\稿件修改\PaperData%20测序\Novel_miRNAs\novel_miRNA_pdf\chr10_126.pdf) | chr10:43471238..43471297:+ | 2.1 | 2 +/- 5% | yes | uggcuggcugcuccgggcacu |
| [**chr18_7818**](file:///G:\论文撰写\Processing\稿件修改\PaperData%20测序\Novel_miRNAs\novel_miRNA_pdf\chr18_7818.pdf) | chr18:58237708..58237772:+ | 2.1 | 2 +/- 5% | yes | acagcucugaugaucucuaugu |
| [**chr4_17916**](file:///G:\论文撰写\Processing\稿件修改\PaperData%20测序\Novel_miRNAs\novel_miRNA_pdf\chr4_17916.pdf) | chr4:103422118..103422180:+ | 2.1 | 2 +/- 5% | yes | caaaaacugcaauuacuuuugc |
| [**chr11_1178**](file:///G:\论文撰写\Processing\稿件修改\PaperData%20测序\Novel_miRNAs\novel_miRNA_pdf\chr11_1178.pdf) | chr11:65503086..65503192:+ | 2.1 | 2 +/- 5% | yes | caggcagcuguuaacag |
| [**chr14_4469**](file:///G:\论文撰写\Processing\稿件修改\PaperData%20测序\Novel_miRNAs\novel_miRNA_pdf\chr14_4469.pdf) | chr14:19302175..19302210:- | 2 | 2 +/- 5% | no | cgcacuugcggccccggc |
| [**chr14_4508**](file:///G:\论文撰写\Processing\稿件修改\PaperData%20测序\Novel_miRNAs\novel_miRNA_pdf\chr14_4508.pdf) | chr14:21429711..21429788:- | 1.9 | 9 +/- 8% | yes | gaguucugggcuguggugugcu |
| [**chr2_13918**](file:///G:\论文撰写\Processing\稿件修改\PaperData%20测序\Novel_miRNAs\novel_miRNA_pdf\chr2_13918.pdf) | chr2:71526828..71526914:+ | 1.9 | 9 +/- 8% | yes | auccuagcuugccugagacug |
| [**chr2_14458**](file:///G:\论文撰写\Processing\稿件修改\PaperData%20测序\Novel_miRNAs\novel_miRNA_pdf\chr2_14458.pdf) | chr2:191488034..191488077:+ | 1.9 | 9 +/- 8% | yes | agauauugggcuguacc |
| [**chr10_464**](file:///G:\论文撰写\Processing\稿件修改\PaperData%20测序\Novel_miRNAs\novel_miRNA_pdf\chr10_464.pdf) | chr10:133242125..133242211:+ | 1.9 | 9 +/- 8% | yes | uuagggcccuggcuccauc |
| [**chr1_9701**](file:///G:\论文撰写\Processing\稿件修改\PaperData%20测序\Novel_miRNAs\novel_miRNA_pdf\chr1_9701.pdf) | chr1:156887614..156887687:+ | 1.8 | 9 +/- 8% | yes | uccccaguacccccacca |
| [**chr16_5652**](file:///G:\论文撰写\Processing\稿件修改\PaperData%20测序\Novel_miRNAs\novel_miRNA_pdf\chr16_5652.pdf) | chr16:12500703..12500776:+ | 1.8 | 9 +/- 8% | yes | agcuugucggaugugcagauuu |
| [**chr6_21972**](file:///G:\论文撰写\Processing\稿件修改\PaperData%20测序\Novel_miRNAs\novel_miRNA_pdf\chr6_21972.pdf) | chr6:90945216..90945273:- | 1.8 | 9 +/- 8% | yes | caaaaacugcagcuacuuuugc |
| [**chr11_1607**](file:///G:\论文撰写\Processing\稿件修改\PaperData%20测序\Novel_miRNAs\novel_miRNA_pdf\chr11_1607.pdf) | chr11:17276489..17276543:- | 1.7 | 9 +/- 8% | no | aggagggacguggcgcucugaga |
| [**chrX_26398**](file:///G:\论文撰写\Processing\稿件修改\PaperData%20测序\Novel_miRNAs\novel_miRNA_pdf\chrX_26398.pdf) | chrX:49200236..49200296:+ | 1.7 | 9 +/- 8% | no | cggcgggggcggcgcgcg |
| [**chr16_5739**](file:///G:\论文撰写\Processing\稿件修改\PaperData%20测序\Novel_miRNAs\novel_miRNA_pdf\chr16_5739.pdf) | chr16:28259172..28259219:+ | 1.7 | 9 +/- 8% | yes | cccccggcuccuccaccc |
| [**chr3_16572**](file:///G:\论文撰写\Processing\稿件修改\PaperData%20测序\Novel_miRNAs\novel_miRNA_pdf\chr3_16572.pdf) | chr3:193997266..193997319:+ | 1.7 | 9 +/- 8% | yes | cuuacuggauugugggga |
| [**chr2_14955**](file:///G:\论文撰写\Processing\稿件修改\PaperData%20测序\Novel_miRNAs\novel_miRNA_pdf\chr2_14955.pdf) | chr2:71706009..71706084:- | 1.7 | 9 +/- 8% | yes | agugcuuggcugaggagcu |
| [**chr5_20388**](file:///G:\论文撰写\Processing\稿件修改\PaperData%20测序\Novel_miRNAs\novel_miRNA_pdf\chr5_20388.pdf) | chr5:139273892..139273949:- | 1.7 | 9 +/- 8% | yes | uuggacucccaucccaucucuu |
| [**chr6_21674**](file:///G:\论文撰写\Processing\稿件修改\PaperData%20测序\Novel_miRNAs\novel_miRNA_pdf\chr6_21674.pdf) | chr6:28587793..28587840:- | 1.6 | 9 +/- 8% | yes | uccgugacuaaacaacugagg |
| [**chr10_271**](file:///G:\论文撰写\Processing\稿件修改\PaperData%20测序\Novel_miRNAs\novel_miRNA_pdf\chr10_271.pdf) | chr10:80486625..80486704:+ | 1.6 | 9 +/- 8% | yes | accacgaggaagagagg |
| [**chr6_21319**](file:///G:\论文撰写\Processing\稿件修改\PaperData%20测序\Novel_miRNAs\novel_miRNA_pdf\chr6_21319.pdf) | chr6:135239184..135239241:+ | 1.5 | 9 +/- 8% | yes | caaaacuggcaauuacuuuug |
| [**chr13_3447**](file:///G:\论文撰写\Processing\稿件修改\PaperData%20测序\Novel_miRNAs\novel_miRNA_pdf\chr13_3447.pdf) | chr13:49978708..49978769:+ | 1.5 | 9 +/- 8% | yes | cagauucuggauuucuacc |
| [**chr17_6969**](file:///G:\论文撰写\Processing\稿件修改\PaperData%20测序\Novel_miRNAs\novel_miRNA_pdf\chr17_6969.pdf) | chr17:78140754..78140817:+ | 1.5 | 9 +/- 8% | yes | aagcagcgccugucgcaacucgcc |
| [**chr17_7557**](file:///G:\论文撰写\Processing\稿件修改\PaperData%20测序\Novel_miRNAs\novel_miRNA_pdf\chr17_7557.pdf) | chr17:74359883..74359924:- | 1.5 | 9 +/- 8% | yes | cccccggcuccuccaccu |
| [**chr19_8631**](file:///G:\论文撰写\Processing\稿件修改\PaperData%20测序\Novel_miRNAs\novel_miRNA_pdf\chr19_8631.pdf) | chr19:13836230..13836310:- | 1.4 | 9 +/- 8% | no | uggcucaguucagcaggaacagg |
| [**chr20_11495**](file:///G:\论文撰写\Processing\稿件修改\PaperData%20测序\Novel_miRNAs\novel_miRNA_pdf\chr20_11495.pdf) | chr20:1855349..1855408:+ | 1.4 | 9 +/- 8% | no | aagguggagaagcugacc |
| [**chr7_22291**](file:///G:\论文撰写\Processing\稿件修改\PaperData%20测序\Novel_miRNAs\novel_miRNA_pdf\chr7_22291.pdf) | chr7:1359873..1359951:+ | 1.4 | 9 +/- 8% | no | accaggagggaggugggg |
| [**chr2_13940**](file:///G:\论文撰写\Processing\稿件修改\PaperData%20测序\Novel_miRNAs\novel_miRNA_pdf\chr2_13940.pdf) | chr2:74198636..74198678:+ | 1.4 | 9 +/- 8% | yes | cggucuauggcugcgac |
| [**chr17_6937**](file:///G:\论文撰写\Processing\稿件修改\PaperData%20测序\Novel_miRNAs\novel_miRNA_pdf\chr17_6937.pdf) | chr17:73878008..73878061:+ | 1.4 | 9 +/- 8% | yes | agagggacggccggggg |
| [**chr6_21601**](file:///G:\论文撰写\Processing\稿件修改\PaperData%20测序\Novel_miRNAs\novel_miRNA_pdf\chr6_21601.pdf) | chr6:27209873..27209920:- | 1.4 | 9 +/- 8% | yes | aggauuugaaccugcgcg |
| [**chr19_8383**](file:///G:\论文撰写\Processing\稿件修改\PaperData%20测序\Novel_miRNAs\novel_miRNA_pdf\chr19_8383.pdf) | chr19:45341553..45341597:+ | 1.4 | 9 +/- 8% | yes | cgcgcgcgcguguggug |
| [**chr9_25608**](file:///G:\论文撰写\Processing\稿件修改\PaperData%20测序\Novel_miRNAs\novel_miRNA_pdf\chr9_25608.pdf) | chr9:91031568..91031629:- | 1.4 | 9 +/- 8% | yes | caaaaacugcauuacuuuugu |
| [**chr17_7086**](file:///G:\论文撰写\Processing\稿件修改\PaperData%20测序\Novel_miRNAs\novel_miRNA_pdf\chr17_7086.pdf) | chr17:7835454..7835515:- | 1.3 | 9 +/- 8% | yes | ucggucccuaacccccuccggac |
| [**chr1_9313**](file:///G:\论文撰写\Processing\稿件修改\PaperData%20测序\Novel_miRNAs\novel_miRNA_pdf\chr1_9313.pdf) | chr1:62101716..62101792:+ | 1.2 | 9 +/- 8% | yes | ucaaauccugucugacccu |
| [**chr10_41**](file:///G:\论文撰写\Processing\稿件修改\PaperData%20测序\Novel_miRNAs\novel_miRNA_pdf\chr10_41.pdf) | chr10:15169102..15169173:+ | 1.2 | 9 +/- 8% | no | ucugccuggggcucggcucu |
| [**chr6_20661**](file:///G:\论文撰写\Processing\稿件修改\PaperData%20测序\Novel_miRNAs\novel_miRNA_pdf\chr6_20661.pdf) | chr6:18571809..18571865:+ | 1.2 | 9 +/- 8% | yes | caaaacuggcaauuacuuuug |
| [**chr5_20490**](file:///G:\论文撰写\Processing\稿件修改\PaperData%20测序\Novel_miRNAs\novel_miRNA_pdf\chr5_20490.pdf) | chr5:168560903..168560965:- | 1.2 | 9 +/- 8% | yes | agcagcauuguacagggcuauga |
| [**chr3_16325**](file:///G:\论文撰写\Processing\稿件修改\PaperData%20测序\Novel_miRNAs\novel_miRNA_pdf\chr3_16325.pdf) | chr3:139496495..139496552:+ | 1.1 | 9 +/- 8% | yes | uaaaccuguauuuuuuga |
| [**chr1_10878**](file:///G:\论文撰写\Processing\稿件修改\PaperData%20测序\Novel_miRNAs\novel_miRNA_pdf\chr1_10878.pdf) | chr1:145720611..145720664:- | 1.1 | 9 +/- 8% | yes | aaggucagcucugagcuccac |
| [**chr8_24737**](file:///G:\论文撰写\Processing\稿件修改\PaperData%20测序\Novel_miRNAs\novel_miRNA_pdf\chr8_24737.pdf) | chr8:140528687..140528756:- | 1.1 | 9 +/- 8% | no | aaacugggcauagcuguacuuuu |
| [**chr20_12140**](file:///G:\论文撰写\Processing\稿件修改\PaperData%20测序\Novel_miRNAs\novel_miRNA_pdf\chr20_12140.pdf) | chr20:38429666..38429739:- | 1.1 | 9 +/- 8% | yes | acccuggagcuuucccacaccu |
| [**chr5_19846**](file:///G:\论文撰写\Processing\稿件修改\PaperData%20测序\Novel_miRNAs\novel_miRNA_pdf\chr5_19846.pdf) | chr5:33712739..33712782:- | 1.1 | 9 +/- 8% | yes | aguacucaagaggcugaagagu |
| [**chr4_17499**](file:///G:\论文撰写\Processing\稿件修改\PaperData%20测序\Novel_miRNAs\novel_miRNA_pdf\chr4_17499.pdf) | chr4:1735491..1735562:+ | 1.1 | 9 +/- 8% | yes | agcccaggaugaaacucugaca |
| [**chr17_6784**](file:///G:\论文撰写\Processing\稿件修改\PaperData%20测序\Novel_miRNAs\novel_miRNA_pdf\chr17_6784.pdf) | chr17:42514267..42514330:+ | 1.1 | 9 +/- 8% | no | uuuugugucucccauuccccaga |
| [**chr13_3448**](file:///G:\论文撰写\Processing\稿件修改\PaperData%20测序\Novel_miRNAs\novel_miRNA_pdf\chr13_3448.pdf) | chr13:49978750..49978799:+ | 0.9 | 0 +/- 0% | yes | cagauucuggauuucuacc |
| [**chrX_26226**](file:///G:\论文撰写\Processing\稿件修改\PaperData%20测序\Novel_miRNAs\novel_miRNA_pdf\chrX_26226.pdf) | chrX:7187376..7187445:+ | 0.9 | 0 +/- 0% | yes | auccuagcuugccugagacug |
| [**chr20_12169**](file:///G:\论文撰写\Processing\稿件修改\PaperData%20测序\Novel_miRNAs\novel_miRNA_pdf\chr20_12169.pdf) | chr20:50242515..50242571:- | 0.9 | 0 +/- 0% | no | aggaggaagguggggaca |
| [**chr8_24165**](file:///G:\论文撰写\Processing\稿件修改\PaperData%20测序\Novel_miRNAs\novel_miRNA_pdf\chr8_24165.pdf) | chr8:144328774..144328835:+ | 0.8 | 0 +/- 0% | yes | gcucagagaggucuggaccugg |
| [**chr18_7779**](file:///G:\论文撰写\Processing\稿件修改\PaperData%20测序\Novel_miRNAs\novel_miRNA_pdf\chr18_7779.pdf) | chr18:45331465..45331525:+ | 0.8 | 0 +/- 0% | yes | cacgugaaacccugucugua |
| [**chr1_10382**](file:///G:\论文撰写\Processing\稿件修改\PaperData%20测序\Novel_miRNAs\novel_miRNA_pdf\chr1_10382.pdf) | chr1:23870826..23870876:- | 0.8 | 0 +/- 0% | no | agagcuuaugagggagga |
| [**chr16_6079**](file:///G:\论文撰写\Processing\稿件修改\PaperData%20测序\Novel_miRNAs\novel_miRNA_pdf\chr16_6079.pdf) | chr16:3152609..3152672:- | 0.8 | 0 +/- 0% | no | uggucuagggguaugaucu |
| [**chr6_21243**](file:///G:\论文撰写\Processing\稿件修改\PaperData%20测序\Novel_miRNAs\novel_miRNA_pdf\chr6_21243.pdf) | chr6:113704865..113704915:+ | 0.8 | 0 +/- 0% | yes | aaaagcugaguugagagggg |
| [**chr21_12885**](file:///G:\论文撰写\Processing\稿件修改\PaperData%20测序\Novel_miRNAs\novel_miRNA_pdf\chr21_12885.pdf) | chr21:9366315..9366359:+ | 0.8 | 0 +/- 0% | no | ucucugggccugugucuu |
| [**chr5_20573**](file:///G:\论文撰写\Processing\稿件修改\PaperData%20测序\Novel_miRNAs\novel_miRNA_pdf\chr5_20573.pdf) | chr5:181234526..181234576:- | 0.7 | 0 +/- 0% | yes | guggagcucugggccugau |
| [**chr2_15044**](file:///G:\论文撰写\Processing\稿件修改\PaperData%20测序\Novel_miRNAs\novel_miRNA_pdf\chr2_15044.pdf) | chr2:90013419..90013480:- | 0.7 | 0 +/- 0% | no | cagcacuguggguacuuga |
| [**chr5_19326**](file:///G:\论文撰写\Processing\稿件修改\PaperData%20测序\Novel_miRNAs\novel_miRNA_pdf\chr5_19326.pdf) | chr5:119329386..119329439:+ | 0.7 | 0 +/- 0% | no | uguggugaugacagaccugagc |
| [**chr4_18811**](file:///G:\论文撰写\Processing\稿件修改\PaperData%20测序\Novel_miRNAs\novel_miRNA_pdf\chr4_18811.pdf) | chr4:184193748..184193803:- | 0.7 | 0 +/- 0% | yes | uuuguagguaaauucugc |
| [**chr7_23422**](file:///G:\论文撰写\Processing\稿件修改\PaperData%20测序\Novel_miRNAs\novel_miRNA_pdf\chr7_23422.pdf) | chr7:109945214..109945292:- | 0.7 | 0 +/- 0% | yes | aaaagaacuuugaagccc |
| [**chr8_23778**](file:///G:\论文撰写\Processing\稿件修改\PaperData%20测序\Novel_miRNAs\novel_miRNA_pdf\chr8_23778.pdf) | chr8:27433413..27433480:+ | 0.6 | 0 +/- 0% | no | uuggcuggucucugcuccgcag |
| [**chr5_20392**](file:///G:\论文撰写\Processing\稿件修改\PaperData%20测序\Novel_miRNAs\novel_miRNA_pdf\chr5_20392.pdf) | chr5:140175002..140175058:- | 0.6 | 0 +/- 0% | yes | uugcagucggcgcagucgg |
| [**chr11_1435**](file:///G:\论文撰写\Processing\稿件修改\PaperData%20测序\Novel_miRNAs\novel_miRNA_pdf\chr11_1435.pdf) | chr11:120336427..120336479:+ | 0.6 | 0 +/- 0% | no | ccucccggaccagggcuucc |
| [**chr2_14027**](file:///G:\论文撰写\Processing\稿件修改\PaperData%20测序\Novel_miRNAs\novel_miRNA_pdf\chr2_14027.pdf) | chr2:89168473..89168534:+ | 0.6 | 0 +/- 0% | no | cagcacuguggguacuuga |
| [**chr2_14961**](file:///G:\论文撰写\Processing\稿件修改\PaperData%20测序\Novel_miRNAs\novel_miRNA_pdf\chr2_14961.pdf) | chr2:73070420..73070496:- | 0.6 | 0 +/- 0% | yes | aaaagaacuuugaaggaag |
| [**chr14_4698**](file:///G:\论文撰写\Processing\稿件修改\PaperData%20测序\Novel_miRNAs\novel_miRNA_pdf\chr14_4698.pdf) | chr14:60092006..60092078:- | 0.6 | 0 +/- 0% | yes | ggggacgacgggagucgcucgugc |
| [**chr3_17425**](file:///G:\论文撰写\Processing\稿件修改\PaperData%20测序\Novel_miRNAs\novel_miRNA_pdf\chr3_17425.pdf) | chr3:186270691..186270762:- | 0.6 | 0 +/- 0% | no | ccuggacuuggggucagaag |
| [**chr15_5183**](file:///G:\论文撰写\Processing\稿件修改\PaperData%20测序\Novel_miRNAs\novel_miRNA_pdf\chr15_5183.pdf) | chr15:89217792..89217829:+ | 0.6 | 0 +/- 0% | yes | ucaaaaacacggcucucug |
| [**chr8_24635**](file:///G:\论文撰写\Processing\稿件修改\PaperData%20测序\Novel_miRNAs\novel_miRNA_pdf\chr8_24635.pdf) | chr8:119886268..119886337:- | 0.6 | 0 +/- 0% | yes | aggacuugugauuggagu |
| [**chr1_9694**](file:///G:\论文撰写\Processing\稿件修改\PaperData%20测序\Novel_miRNAs\novel_miRNA_pdf\chr1_9694.pdf) | chr1:156137678..156137769:+ | 0.5 | 0 +/- 0% | yes | cgcucagugacugugguugagga |
| [**chr9_25616**](file:///G:\论文撰写\Processing\稿件修改\PaperData%20测序\Novel_miRNAs\novel_miRNA_pdf\chr9_25616.pdf) | chr9:93238386..93238430:- | 0.5 | 0 +/- 0% | yes | aucaagaacacagcucuc |
| [**chr9_25417**](file:///G:\论文撰写\Processing\稿件修改\PaperData%20测序\Novel_miRNAs\novel_miRNA_pdf\chr9_25417.pdf) | chr9:30903692..30903771:- | 0.5 | 0 +/- 0% | yes | aguugaaggucuuaaga |
| [**chr3_16803**](file:///G:\论文撰写\Processing\稿件修改\PaperData%20测序\Novel_miRNAs\novel_miRNA_pdf\chr3_16803.pdf) | chr3:48635405..48635458:- | 0.4 | 0 +/- 0% | no | uuccggagcguagcggccucuagc |
| [**chr7_22396**](file:///G:\论文撰写\Processing\稿件修改\PaperData%20测序\Novel_miRNAs\novel_miRNA_pdf\chr7_22396.pdf) | chr7:25264819..25264879:+ | 0.4 | 0 +/- 0% | yes | ccauugaugaucguucuucu |
| [**chr20_11949**](file:///G:\论文撰写\Processing\稿件修改\PaperData%20测序\Novel_miRNAs\novel_miRNA_pdf\chr20_11949.pdf) | chr20:7439074..7439127:- | 0.4 | 0 +/- 0% | yes | auuguuugaguguugaaua |
| [**chr5_20509**](file:///G:\论文撰写\Processing\稿件修改\PaperData%20测序\Novel_miRNAs\novel_miRNA_pdf\chr5_20509.pdf) | chr5:172404832..172404894:- | 0.4 | 0 +/- 0% | yes | ucagacacagguauggcuggcucc |
| [**chr12_2611**](file:///G:\论文撰写\Processing\稿件修改\PaperData%20测序\Novel_miRNAs\novel_miRNA_pdf\chr12_2611.pdf) | chr12:104571558..104571650:+ | 0.4 | 0 +/- 0% | no | ucugaugaugaaguuugucugacu |
| [**chr10_19**](file:///G:\论文撰写\Processing\稿件修改\PaperData%20测序\Novel_miRNAs\novel_miRNA_pdf\chr10_19.pdf) | chr10:5687107..5687172:+ | 0.4 | 0 +/- 0% | yes | uuugucaguacauguuaauggu |
| [**chr2_14828**](file:///G:\论文撰写\Processing\稿件修改\PaperData%20测序\Novel_miRNAs\novel_miRNA_pdf\chr2_14828.pdf) | chr2:40890485..40890542:- | 0.4 | 0 +/- 0% | yes | cccaggguggcugcugca |
| [**chr2_14944**](file:///G:\论文撰写\Processing\稿件修改\PaperData%20测序\Novel_miRNAs\novel_miRNA_pdf\chr2_14944.pdf) | chr2:69520045..69520106:- | 0.3 | 0 +/- 0% | yes | uucauuugccucccagccuaca |
| [**chr9_25244**](file:///G:\论文撰写\Processing\稿件修改\PaperData%20测序\Novel_miRNAs\novel_miRNA_pdf\chr9_25244.pdf) | chr9:129503951..129504013:+ | 0.3 | 0 +/- 0% | yes | ugauaucaggggcuugcagccug |
| [**chr3_17078**](file:///G:\论文撰写\Processing\稿件修改\PaperData%20测序\Novel_miRNAs\novel_miRNA_pdf\chr3_17078.pdf) | chr3:111026350..111026419:- | 0.3 | 0 +/- 0% | yes | gaaucugaaaaaaaucugaaa |
| [**chr12_2273**](file:///G:\论文撰写\Processing\稿件修改\PaperData%20测序\Novel_miRNAs\novel_miRNA_pdf\chr12_2273.pdf) | chr12:29562570..29562641:+ | 0.3 | 0 +/- 0% | yes | acuggauuuggagucagaagcc |
| [**chr14_4695**](file:///G:\论文撰写\Processing\稿件修改\PaperData%20测序\Novel_miRNAs\novel_miRNA_pdf\chr14_4695.pdf) | chr14:60036399..60036454:- | 0.3 | 0 +/- 0% | yes | ucucuggucuggaagauuccu |
| [**chr19_8618**](file:///G:\论文撰写\Processing\稿件修改\PaperData%20测序\Novel_miRNAs\novel_miRNA_pdf\chr19_8618.pdf) | chr19:10928439..10928503:- | 0.2 | 0 +/- 0% | yes | ugaccggucucuguccucagc |
| [**chr17_6973**](file:///G:\论文撰写\Processing\稿件修改\PaperData%20测序\Novel_miRNAs\novel_miRNA_pdf\chr17_6973.pdf) | chr17:79504510..79504583:+ | 0.2 | 0 +/- 0% | yes | ucuggggcugcagcaacucu |
| [**chr18_8035**](file:///G:\论文撰写\Processing\稿件修改\PaperData%20测序\Novel_miRNAs\novel_miRNA_pdf\chr18_8035.pdf) | chr18:47357250..47357286:- | 0.2 | 0 +/- 0% | no | aagagacaugagaggua |
| [**chr1_10414**](file:///G:\论文撰写\Processing\稿件修改\PaperData%20测序\Novel_miRNAs\novel_miRNA_pdf\chr1_10414.pdf) | chr1:28581030..28581092:- | 0.2 | 0 +/- 0% | no | uuggcccuuaucgaagcugcagc |
| [**chr9_25480**](file:///G:\论文撰写\Processing\稿件修改\PaperData%20测序\Novel_miRNAs\novel_miRNA_pdf\chr9_25480.pdf) | chr9:37885782..37885841:- | 0 | 0 +/- 0% | yes | auugcguuggccacuuuacu |
| [**chr9_25804**](file:///G:\论文撰写\Processing\稿件修改\PaperData%20测序\Novel_miRNAs\novel_miRNA_pdf\chr9_25804.pdf) | chr9:133196464..133196531:- | 0 | 0 +/- 0% | yes | uauggauguggaaguaucuuuu |
| [**chr9_25000**](file:///G:\论文撰写\Processing\稿件修改\PaperData%20测序\Novel_miRNAs\novel_miRNA_pdf\chr9_25000.pdf) | chr9:74209396..74209474:+ | 0 | 0 +/- 0% | yes | gaagagaacuuugaaaagag |
| [**chr1_9814**](file:///G:\论文撰写\Processing\稿件修改\PaperData%20测序\Novel_miRNAs\novel_miRNA_pdf\chr1_9814.pdf) | chr1:171591080..171591150:+ | 0 | 0 +/- 0% | yes | ugguaggagcuaucagaacuuagu |
| [**chr1_10289**](file:///G:\论文撰写\Processing\稿件修改\PaperData%20测序\Novel_miRNAs\novel_miRNA_pdf\chr1_10289.pdf) | chr1:8180098..8180166:- | 0 | 0 +/- 0% | yes | aaauauuauuuagaacuagc |
| [**chr10_139**](file:///G:\论文撰写\Processing\稿件修改\PaperData%20测序\Novel_miRNAs\novel_miRNA_pdf\chr10_139.pdf) | chr10:48614184..48614244:+ | 0 | 0 +/- 0% | yes | aguguuuucacaagguagu |
| [**chr2_14913**](file:///G:\论文撰写\Processing\稿件修改\PaperData%20测序\Novel_miRNAs\novel_miRNA_pdf\chr2_14913.pdf) | chr2:61509653..61509730:- | 0 | 0 +/- 0% | yes | uuugggcuuuuucagauuuuggaau |
| [**chr21_13013**](file:///G:\论文撰写\Processing\稿件修改\PaperData%20测序\Novel_miRNAs\novel_miRNA_pdf\chr21_13013.pdf) | chr21:42321289..42321366:+ | 0 | 0 +/- 0% | yes | ucucccuuccugcccugg |
| [**chr12_2382**](file:///G:\论文撰写\Processing\稿件修改\PaperData%20测序\Novel_miRNAs\novel_miRNA_pdf\chr12_2382.pdf) | chr12:53463161..53463225:+ | 0 | 0 +/- 0% | yes | ugggcuguggugccgugaccuuug |
| [**chr6_21534**](file:///G:\论文撰写\Processing\稿件修改\PaperData%20测序\Novel_miRNAs\novel_miRNA_pdf\chr6_21534.pdf) | chr6:19096152..19096217:- | 0 | 0 +/- 0% | yes | aaggaaaaugaaguuca |
